# Climate Policy Reduces Racial Disparities in Air Pollution from Transportation and Power Generation

**DOI:** 10.1021/acs.est.4c03719

**Published:** 2024-11-26

**Authors:** Katherine H. Jordan, Luke R. Dennin, Peter J. Adams, Paulina Jaramillo, Nicholas Z. Muller

**Affiliations:** 1 Engineering and Public Policy, Carnegie Mellon University, 5000 Forbes Ave., Pittsburgh, Pennsylvania 15213, United States; 2 Civil and Environmental Engineering, Carnegie Mellon University, 5000 Forbes Ave., Pittsburgh, Pennsylvania 15213, United States; 3 Tepper School of Business, Carnegie Mellon University, 5000 Forbes Ave., Pittsburgh, Pennsylvania 15213, United States; 4 National Bureau of Economic Research, 1050 Massachusetts Avenue, Cambridge, Massachusetts 02138, United States

**Keywords:** energy system modeling, decarbonization, air quality, equity, environmental justice

## Abstract

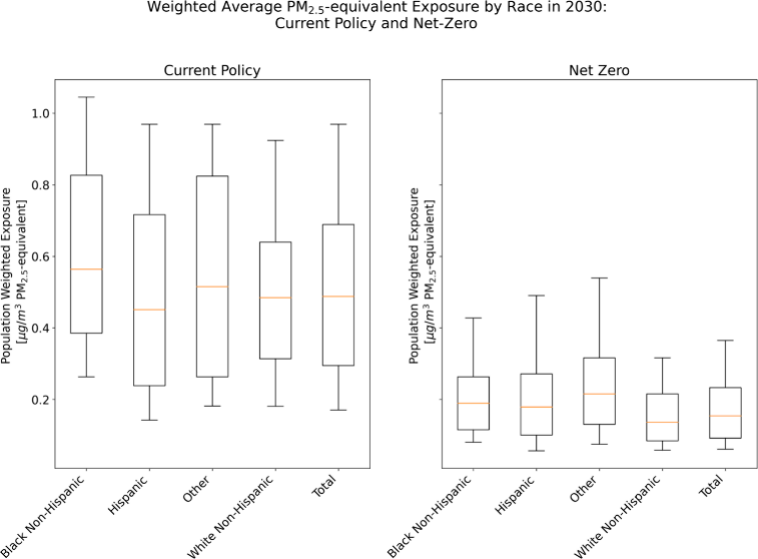

Energy system optimization models facilitate analyses on a national or regional scale. However, understanding the impacts of climate policy on specific populations requires a much higher spatial resolution. Here, we link an energy system optimization model to an integrated assessment model via an emission downscaling algorithm, translating air pollution emissions from nine U.S. regions to U.S. counties. We simulate the impacts of six distinct policy scenarios, including a current policy and a 2050 net-zero target, on NO_*x*_, SO_2_, and PM_2.5_ emissions from on-road transportation and electricity generation. We compare different policies based on their ability to reduce emission exposure and exposure disparity across racial groups, allowing decision-makers to assess the air pollution impacts of various policy instruments more holistically. Modeled policies include a clean electricity standard, an on-road ICE vehicle ban, a carbon tax, and a scenario that reaches net-zero GHG emissions by 2050. While exposure and disparities decrease in all scenarios, our results reveal persistent disparities until at least 2040, particularly for Black non-Hispanic Americans. Our estimates of avoided deaths due to air pollution emphasize the importance of policy timing, showing that thousands of lives can be saved by taking action in the near-term.

## Abstract 2

Reducing greenhouse gas emissions from cars, trucks, and electricity generation decreases racial disparities in air pollution exposure, but historical inequities persist for decades, emphasizing the need for near-term action.

## 1 Introduction

Many countries, including the United States, are adopting policies to reduce greenhouse gas emissions to mitigate the worst impacts of anthropogenic climate change.^1,2^ These policies stand to transform economic systems fundamentally.^3^ The ramifications of such a large-scale change are likely to manifest unequally across society.^4−6^ As such, governments must focus on ensuring an equitable energy transition. For example, the Justice40 initiative sets a goal that 40% of overall benefits from certain federal investments will go to marginalized and underserved communities.^7^ Historically, regulatory impact assessments have said nothing about environmental justice goals or made brief qualitative statements. This analysis aims to inform policy design in this domain by synthesizing results from the electric power generation and on-road transportation sectors of an economy-wide energy system model coupled with high-resolution health impact analyses to quantify environmental justice outcomes.

The literature describes three primary areas of equity: procedural, recognitional, and distributional.^6,8,9^ This paper focuses on distributional equity, the fair distribution of benefits across all stakeholders.^8^ Through this lens, we explore how different decarbonization policies affect racial groups’ exposure to air pollution, expanding the literature that explores different metrics to score energy transition equity outcomes.^10−12^

Our equity-focused analysis is motivated by three facts. First, over 100 million U.S. residents live in counties that do not meet the National Ambient Air Quality Standards that govern air pollution concentrations.^13^ Second, air pollution is concentrated in communities of color and low-income communities.^14−19^ Third, exposure to air pollution is associated with acute and chronic health effects, including premature mortality.^20−23^ Numerous studies show that climate policy produces substantial cobenefits from reductions in air pollution.^24−28^ Energy system optimization models (ESOMs) are one tool researchers and policymakers can use to study changes in greenhouse gas and air pollution emissions resulting from policy instruments and technological advancement. Researchers have used ESOMs to explore national emissions in the absence of new federal climate policy,^29^ opportunities for power sector decarbonization,^30,31^ pathways to achieving net-zero emissions,^32,33^ and the impact of carbon taxes on emissions and technology deployment^34−36^ and to assess realistic policy instruments.^37^

These studies offer insights into the aggregate consequences of energy system decarbonization. However, studies that use national or regional scale models are ill-suited to assess distributional equity outcomes.^38,39^ The air quality models that researchers use to understand spatially resolved concentration changes and health outcomes require spatially disaggregated emission inputs.^40^ Modelers using ESOMs must make trade-offs between spatial and temporal resolution, technology detail, and the ability to represent multisector interactions. Increasing any of these improves the realism of the model’s results but may make computational time intractable. However, researchers can explore distributional impacts without increasing the spatial resolution of ESOMs (and, as a result, increasing the computational intensity) by downscaling emissions and performing posterior analyses. In this work, we downscale nitrogen oxide (NO_*x*_), sulfur dioxide (SO_2_), and particulate matter (PM_2.5_) emissions from power plants and on-road transportation vehicles to use as inputs into an air quality model.

Existing work explores the distributional equity implications of different subsectors of the current and near-future energy system. For example, a substantial body of work explores the emission impacts of near-term electric vehicle adoption.^14,41−43^ Holland et al. find, for example, that individuals living in census block groups with a median income greater than $65,000 have positive environmental benefits from electric vehicles, but individuals below this threshold receive negative externalities.^42^ The authors also explore how their findings vary by racial group, reporting that White and Black individuals receive negative externalities, while Hispanic and Asian residents receive positive benefits. Thakrar et al. quantify source-specific PM_2.5_ mortalities from air pollution sources, finding that half of all air pollution deaths in the U.S. are attributed to just five sources, including electricity generation and passenger vehicle use.^44^ Tessum et al.^15^ explore economy-wide exposure disparities for different sources of fine particulate matter (PM_2.5_) in five racial–ethnic groups. Their work found that racial–ethnic minorities are exposed to higher levels of PM_2.5_ in nearly all of the major emission categories.

Notably, these studies do not explore how future policy instruments might change future distributional impacts, but a growing body of work is dedicated to understanding this question. Several U.S.-based analyses assess future changes to air pollution exposure driven by policy change. Goforth and Nock explore future equity impacts from the energy transition, finding that national mandates requiring more than 80% deployment of low-carbon technologies in the power sector achieve equality of air pollution concentrations across demographic groups.^45^ Polonik et al. quantify air pollution-related equity outcomes from climate policy using five heuristic pathways consistent with the U.S. NDC but do not simulate explicit technology changes.^46^ Picciano et al. assess whether scenarios that achieve the same CO_2_ reduction (∼50%) can better reduce PM_2.5_ disparities, finding that limited opportunities exist to further mitigate disparities without deeper decarbonization.^47^

Some studies additionally assess equity outcomes either for global emission changes or changes within a specific region of the U.S. Huang et al. use a coupled climate-energy-health model to simulate the impact of climate policy on air pollution globally, focusing on cross-country inequity.^48^ Zhu et al. use annual air quality simulations to assess environmental justice outcomes in California under an 80% CO_2_ reduction policy case, finding that the distribution of benefits changes depending on technology deployment and fuel use in individual end-use sectors.^49^ Wang et al. and Li et al. both explore the impact of low-carbon transportation policy on air pollution exposure in California.^50,51^ Yu et al. found that zero-emission vehicle adoption in California resulted in reduced air pollution exposure but that traffic-related air pollution disparities remain.^52^

The present study explores air pollution-driven distributional equity outcomes in the U.S. under a range of forward-looking decarbonization policies in multiple economic sectors while explicitly modeling policy-driven changes in technology deployment. In addition to a current policy baseline, we model a clean electricity standard, a ban on new internal combustion engine (ICE) vehicles, a carbon tax, and a scenario that reaches net-zero emissions of GHGs in 2050. We simulate changes in air pollution exposure in the U.S. transportation and electric sectors based on the need to decarbonize these sectors in tandem, as addressed in the prior literature.^41,53,54^

## 2 Materials and Methods

In this study, we use an energy system optimization model to simulate six policy scenarios: a current policy baseline (that includes the U.S. Inflation Reduction Act), a ban on new, on-road internal combustion engine vehicles, a clean electricity standard, a combined ICE ban and clean electricity standard, a carbon tax, and a scenario in which GHG emissions decrease linearly from 2020 levels to net zero in 2050. We then downscale the simulated NO_*x*_, SO_2_, and PM_2.5_ emissions from nine U.S. regions to U.S. counties and run the downscaled emissions through the air pollution module of an integrated assessment model. We assess exposure to air pollution and avoided deaths by race–ethnicity under each of the modeled scenarios.

### 2.1 Model Structure

This work uses the Tools for Energy Model Optimization and Analysis (Temoa), a bottom-up energy system optimization model.^55^ Temoa is well-suited to answering questions about decarbonizing the energy system, as it endogenously optimizes fuel use and technology deployment across the energy economy, representing electricity generation, transportation, commercial and residential buildings, heavy industry, and fuel production and supply. Bottom-up models represent technology-explicit choices and require robust techno-economic characterizations of technologies in all end-use sectors, including capital costs, fixed and variable costs, efficiencies, and emission activities.^56^ Temoa is a linear optimization model that considers particular technologies’ interactions within a defined system. Temoa finds the least-cost solution by optimizing installed capacity and the associated activity while ensuring that the energy produced equals or exceeds the energy consumed. Temoa is similar in functionality to the suite of MARKAL/TIMES models, OSeMOSYS, and MESSAGE.^57−59^ Several studies have identified Temoa as a top open-source macro-energy system model.^60−62^ Some examples of previous work using Temoa include quantifying U.S. energy-related GHGs in the absence of federal climate policy,^29^ exploring the impacts of the U.S. Inflation Reduction Act,^63^ and assessing diverse, near cost-optimal pathways to deep decarbonization in the U.S.^64^ Our GitHub repository contains all model codes.^65^

### 2.2 Database

The database used in this work represents the continental U.S. as nine regions. The modeled time horizon extends from 2020 to 2050 and runs in five-year increments, optimizing the first year in a set and applying the result to each year in a five-year period. The model employs a representative day temporal framework, utilizing hourly resolution for eight representative days in each model year. It includes emission factors for GHGs, SO_2_, NO_*x*_, and PM_2.5_, which vary based on fuel and technology combination.

The Temoa database includes representations of many existing state and federal policies, including up-to-date CAFE standards, California’s zero-emission vehicle standard, the mercury and air toxics standards, state-level renewable portfolio standards, and key provisions of the Inflation Reduction Act (IRA), including tax credits for zero-emission vehicles, zero-emission power generation, carbon capture, and clean hydrogen production. Detailed, sector-level documentation can be found in our GitHub repository.^65^

### 2.3 Downscaling

There are three well-defined methods to spatially downscale power plant siting in the literature.^66^ The first is statistical downscaling, which uses scaling, interpolation, and regression.^67^ Another commonly used method, grow-in-place, assumes that new power plants are constructed where old plants were sited.^68−70^ Last, fundamental-based downscaling, or site-and-grow, uses detailed land-use data sets to understand siting decision processes, including their economic and technical drivers.^66^ Site-and-grow is a more computationally intensive method than grow-in-place, requiring detailed land-use data and additional time and computational power. In this study, we implement a modified grow-in-place algorithm for electricity generation units (EGUs). We follow a similar method for vehicles but combine the model results with a spatial surrogate.

Temoa represents the United States in nine geographic regions, meaning that in its current form, it is ill-suited to understanding the equity implications of technology and policy changes. This work implements a postprocessing algorithm to downscale NO_*x*_, SO_2_, and PM_2.5_ emissions from electricity generation and on-road vehicles. We subsequently run the downscaled results through the atmospheric modeling component of the AP3 integrated assessment model^71^ to quantify changes in emission exposure at the county scale. Temoa simulates NO_*x*_, SO_2_, and primary PM_2.5_ emissions from power plants and on-road vehicles. NO_*x*_ and SO_2_ are precursors to ambient PM_2.5_, which increases mortality risk.^72^ AP3 calculates damages from ambient PM_2.5_ formed by primary PM_2.5_, as well as from NO_*x*_ and SO_2_. We then map these results to racial groups to understand the future distribution of air quality and public health changes under different policy instruments. We develop separate electric and transportation emission algorithms, which we detail below.

#### 2.3.1 Electric Sector Emissions

We draw techno-economic parameters for existing EGUs in the Temoa database from PowerGenome,^73^ which compiles data from the Public Utilities Data Liberation (PUDL) Project.^74^ Temoa does not model individual electric generators; instead, we implement clusters of power plants created by PowerGenome based on the plant heat rate. However, we retain the EIA plant ID and the PUDL unit ID for each EGU in each cluster. We used PUDL, EIA, and eGrid data to map the PowerGenome EGUs to actual EGUs. While this tells us the location of each EGU, Temoa does not report the percent of generation in a cluster that comes from each EGU. We therefore use the aforementioned data sources to determine PUDL unit-level 2020 electric generation data. We sum actual generation by Temoa cluster and then determine the percent of generation attributable to each unit. We assume that the percent of generation from each unit stays constant over time, even if Temoa reports total generation from the cluster increasing or decreasing.

To determine the location of the future capacity, we implement a grow-in-place heuristic. We use data on planned EGUs and EGUs that have retired since 2002 from December 2021’s Form EIA-860 M “Monthly Update to Annual Electric Generator Report” to map existing and planned facilities to Temoa’s capacity.^75^ Additional details on downscaling can be found in the Supporting Information.

#### 2.3.2 Transportation Sector Emissions

While Temoa retains information about the exact location of the electric sector technologies, the same is not true for the transportation sector. As such, we map regional vehicle miles traveled (VMT) to county-level VMT using data from the EPA’s MOtor Vehicle Emission Simulator (MOVES), which estimates county-level VMT by vehicle type for 2023, 2026, and 2032. We map MOVES VMT estimates to Temoa’s 2020–2024, 2025–2029, and 2030–2034 time periods, respectively. For the remaining time periods (2035–2050), we implement MOVES-provided national scaling factors by vehicle type, relative to 2032. For a given technology (i) and county (j) within a region (k), we calculate VMT as
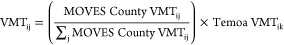


Transportation emissions are then calculated using Temoa’s emissions factors. Temoa considers only emissions from transportation fuel combustion. As a result, our results will underestimate damages from primary PM2.5, as non-exhaust sources (i.e., brake- and tire-wear) are not modeled. While this is a limitation of the study, recent work from Arter et al.^76^ estimates that >70% of premature mortalities from light- and heavy-duty vehicles in the U.S. are attributable to NO_*x*_.

### 2.4 Integrated Assessment Modeling

#### 2.4.1 Air Pollution Modeling

We use the atmospheric modeling component of the AP3 IAM^77,78^ to connect SO_2_, NO_*x*_, and primary PM_2.5_ emissions from EGUs and on-road transportation vehicles to ambient PM_2.5_ concentrations on the margin (i.e., atop the existing baseline). AP3 employs a reduced-complexity framework to model ambient PM_2.5_ in every contiguous U.S. county based on emissions from all domestic sources. These data are provided for 2017 in the EPA’s National Emissions Inventory (NEI).^79^ As such, AP3's nonlinear atmospheric chemistry module relies on relative concentrations based on the 2017 baseline for all years modeled in Temoa. Although future changes in the relative concentrations of relevant pollutants may increase the marginal concentrations associated with SO_2_ and NO_*x*_ emissions, the current state and expected trajectory of emissions suggest that these changes are likely to be limited. See the Supporting Information for a detailed discussion of this topic.

Gaussian plume-based atmospheric modeling predicts speciated pollution concentrations in each receptor location (population-weighted county centroids) from each source location’s emissions. Transportation and EGU emissions are both modeled as being discharged from their county’s population-weighted centroid. However, their effective release heights differ: emissions from the transportation sector are modeled at the ground level, while those from EGUs are modeled using AP3's point source bin for facilities with effective heights (stack height plus plume rise) between 250 and 500 m.^78^ (See the Supporting Information for a discussion of AP3’s modeling based on facility effective heights. Facilities are categorized into three bins based on their effective heights: low (*x* < 250 m), medium (250 m < *x* < 500 m), and tall (*x* > 500 m). Approximately two-thirds of 2017 EGU emissions came from those falling into the medium bin. Because AP3’s tall bin inventory has remained unchanged since the development of the Air Pollution Emission Experiments and Policy analysis model—AP3’s predecessor, along with AP2—most EGUs that would fall into the tall bin default to the medium bin. Relatively few emissions are associated with EGUs in the low bin.) AP3 then models interpollutant chemistry processes, which account for the equilibrium between ammonium, nitrates, and sulfates, to estimate and subsequently aggregate all subspecies of PM_2.5_. The modeled PM_2.5_ concentrations are calibrated using data from the EPA’s Air Quality System monitors.^80^

Using AP3, we estimate the impacts from electric generation and transportation vehicles’ SO_2_, NO_*x*_, and PM_2.5_ emissions for each year from 2020 to 2050. We employ the model to compute marginal concentrations (annual average μg/m^3^ per short ton in every contiguous U.S. county) for each source and pollutant. AP3 adds 1 ton of emissions to the baseline for each source–pollutant pair and estimates marginal impacts by subtracting baseline concentrations from the new concentrations with the marginal ton added. Then, the model is reset to the baseline. This algorithm is repeated for each source and pollutant, and the results of each run differ in where the impacts occur. Last, we multiply total emissions from each source by the respective marginal concentrations for total PM_2.5_ impacts in every county. See the Supporting Information for more details on AP3.

#### 2.4.2 Health Impact Modeling

Temoa provides changes in emissions, and AP3's atmospheric modeling provides changes in exposures, but policymakers and laypeople may find changes in mortality to be a more salient metric. Moreover, susceptibility to the adverse effects of PM_2.5_ varies by subpopulation,^81^ and incorporating health impacts and associated inequities allows us to more holistically examine environmental justice across relevant attributes. Exposures to various pollutants drive several negative health outcomes, but we focus on premature mortality linked to PM_2.5_, which accounts for the majority of local air pollution health damages.^82^ We employ methods from AP3 using a concentration–response function (eq 1) to associate increased PM_2.5_ concentrations with increased mortality risk:
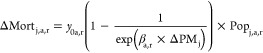
1

*y*_0_ is the baseline mortality rate for each race (r) and age (a).^83^ β is a measure for relative risk associated with a change in PM_2.5_ exposure (ΔPM) in a given county (j).^84^ The function’s output, ΔMort, is the expected premature mortality for each population group driven by the evaluated change in PM_2.5_. Pop is the group-specific population.

Baseline mortality and relative risk are the key health-related inputs to the concentration–response function. There is substantial evidence that people of color experience elevated risk from PM_2.5_ exposure,^15,16,45,85,86^ but data and empirical studies informing our modeling come with caveats and uncertainty. We use 2017 CDC age- and race-specific baseline mortality rates,^83^ but these data are characterized by complex trends such as the Hispanic Paradox. (The Hispanic Paradox is the observation of lower all-cause mortality rates in Hispanic Americans than in non-Hispanic Whites.) Hence, we run sensitivity using all-person age-specific baseline mortality rates, ignoring variation by race. Additionally, the literature conflicts with whether relative risk differs by racial group. Pope et al. find that differences in air pollution-related mortality by race–ethnicity are not statistically significant.^87^ Contrarily, Di et al. find statistically significant differences in risk between racial groups.^81^ Importantly, Di et al.’s analysis is limited to individuals ages 65 and older, but we apply their values to the population of adults aged 30+. To test what these differences would imply for the remainder of the population at risk from PM_2.5_ exposure, we use Di et al.’s estimates of relative risk in one scenario due to the strong relationship they quantify between race–ethnicity and air pollution-related mortality. Otherwise, we simulate mortalities using Krewski et al.’s relative risk estimate, which is constant for all race–ethnicities.^84^ We provide details of these assumptions in the Supporting Information.

As mentioned above, this work simulates emissions of only NO*_x_*, SO_2_, and PM_2.5_. Other pollutants, including ammonia and volatile organic compounds, are not represented in Temoa in its current form. As a result, our estimates of damages and changes to mortality are conservative.

We used population projections from the Socioeconomic Data and Applications Center. This data set provides county-level population projections by sex, race, and age out to 2100.^88^ This data set projects U.S. population according to the shared socioeconomic pathways (SSPs). The SSPs are scenarios that describe alternative socio-economic trajectories out to 2100.^89^ SSP2 is the most consistent with our input data; therefore, we only use population projections under this pathway.

In the population data set, the U.S. population increases 20% from 2020 to 2050 to ∼402,000,000 people. The population density increases primarily in the eastern and western U.S., with fewer changes in the density in the central states. The country becomes more racially diverse, with the population of white non-Hispanic individuals falling from 60 to 48% of the population by 2050. The Hispanic population increases from 18 to 25%, and the Black non-Hispanic population increases from 13 to 14% of the population. The population of other racial groups increases from 7 to 11%.

### 2.5 Modeled Policies

We explore a range of politically salient policies designed to focus on the transportation and electric sectors, which are highly emitting and increasingly coupled due to vehicle electrification. We compare each policy scenario outlined in Table 1 to a baseline “current policy”, which includes only existing policies, including key provisions from the IRA.

**Table 1:** Modeled Policies

**policy**	**description**
current policy	Only existing policies modeled, including the Inflation Reduction Act
clean electricity standard	Requires 80% clean electricity by 2030.^90^ We allow wind, solar, hydroelectric, nuclear, and fossil generation with carbon capture and storage to contribute to the standard. The CES rises linearly from 80% in 2030 to 100% in 2050 in our simulations.
carbon tax	Carbon tax based on the White House’s estimate of the social cost of carbon.^91^ Rises from approximately $50 to $80 per metric ton CO_2_ over the modeled time horizon.
ICE ban	Ban on light-duty internal combustion engine (ICE) vehicle sales (passenger vehicles, commercial trucks, buses, and medium- and heavy-duty trucks). Requires that at least 80% of light-duty vehicle sales are zero-emission by 2030 and 100% by 2035 and that at least 35% of medium and heavy-duty vehicle (short- and long-haul class 8 trucks, school, passenger, and transit buses) sales are zero-emission by 2030, rising linearly to 100% by 2045. Vehicles already on the road are not affected by this policy.
ICE ban + CES	Clean electricity standard + ICE ban
net-zero	Linear decrease from 2020 GHG emissions to net-zero GHG emissions in 2050. Net zero allows for positive GHG emissions as long as they are offset by carbon dioxide removal technologies, such as direct air capture.

## 3 Results

The six scenarios evaluated led to different trajectories in energy production and use, as shown in Figures S.2–S.4. These technology deployment differences drive differences in emissions, exposure, and disparity outcomes. For example, Figure S.2 shows that fossil-based electricity generation totals 480 TWh in 2030 in the net-zero scenario, while coal and natural gas account for 690 TWh that year in the current policy scenario.

### 3.1 Exposure

**Figure 1:**
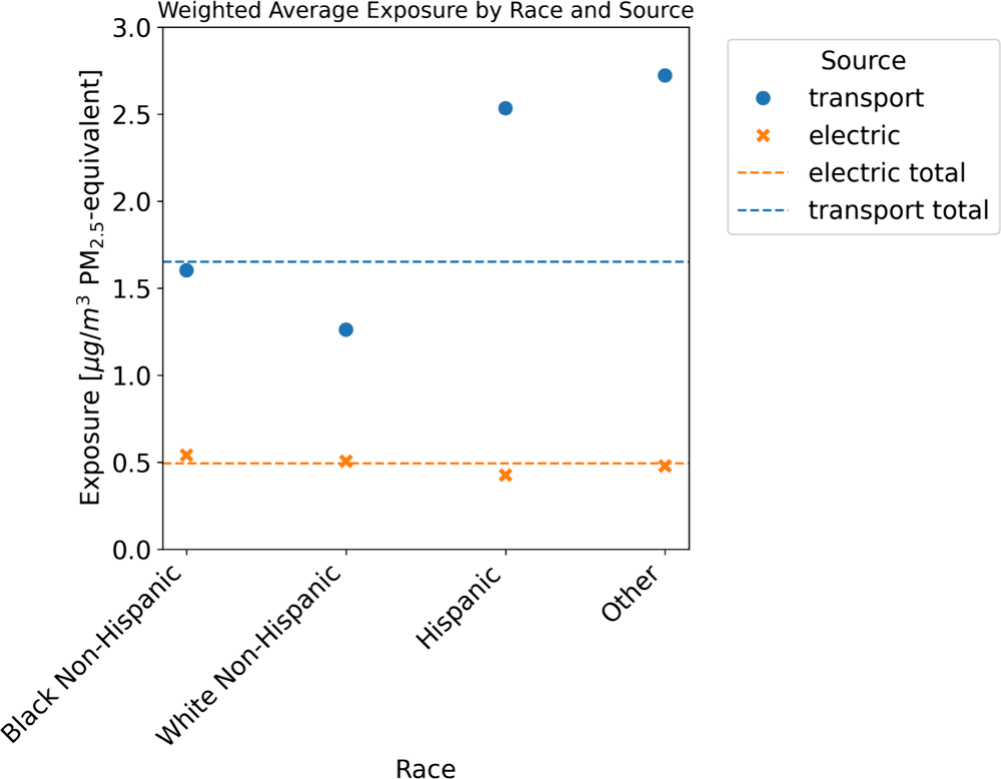
2020 population-weighted average PM_2.5_ exposure by race and source. The “total” lines indicate the overall population-weighted average exposure by source.

In the first time period (2020–2024), the exposure disparity is higher for on-road transportation vehicles than for electricity generation, as illustrated in Figure 1. Disparity is defined as the difference between the population-weighted race–ethnicity-specific exposure and the total population-weighted exposure in a given county. White non-Hispanics are exposed to an average of 0.34, 1.26, and 2.72 μg/m^3^ less pollution from on-road transportation than Black non-Hispanics, Hispanics, and other racial groups, respectively. For EGUs, Hispanics have the lowest population-weighted average exposure, with 0.11, 0.08, and 0.05 μg/m^3^ lower than those of Black non-Hispanics, White non-Hispanics, and other racial groups, respectively. These baseline results largely agree with the broader exposure literature.^92,93^

**Figure 2:**
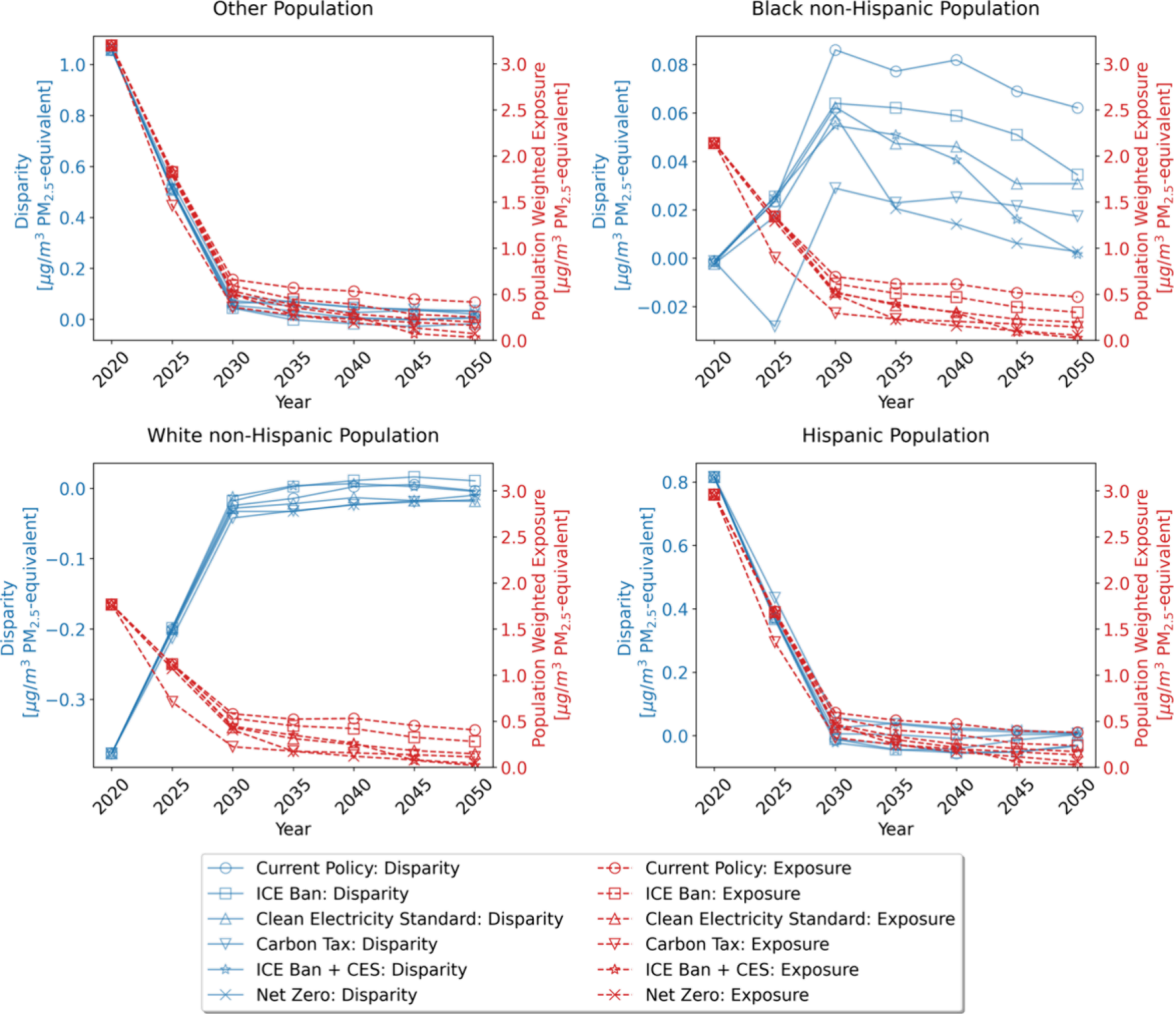
Disparity (left *y*-axis) and population-weighted exposure (right *y*-axis) over time by racial group and policy.

Figure 1 demonstrates existing disparities in population-weighted exposure, particularly for Hispanic and Other Americans, driven by on-road transportation emissions. Over time, exposure decreases under all modeled policies, as demonstrated in Figure 2. Figure 2 plots the population-weighted exposure and disparity in every modeled time period for each population group. Several trends are evident. First, for all groups and all policies, population-weighted exposure decreases over time. For Hispanic and Other Americans, disparity similarly decreases. For Black and White non-Hispanic Americans, the trends are opposite. The disparity for White non-Hispanic Americans trends toward zero, just as Other and Hispanic disparities, but because the initial disparity for White non-Hispanics is negative, this appears as an increase on the plot. Thus, despite the trendline moving in the opposite direction as the Other and Hispanic lines, the White non-Hispanic subplot still indicates progress toward equity. For all racial groups, exposure remains the highest under the current policy and ICE ban scenarios by 2050.

This is not the case for Black non-Hispanic Americans, however. For this group, the exposure disparity in 2020 is zero. While exposure decreases for this group, it does so at a slower rate than for Hispanics and Other Americans, leading to an increase in disparity relative to 2020. The net-zero and ICE ban + CES policies see a return to approximately zero disparity by 2050, but disparity remains under the other modeled policies.

**Figure 3:**
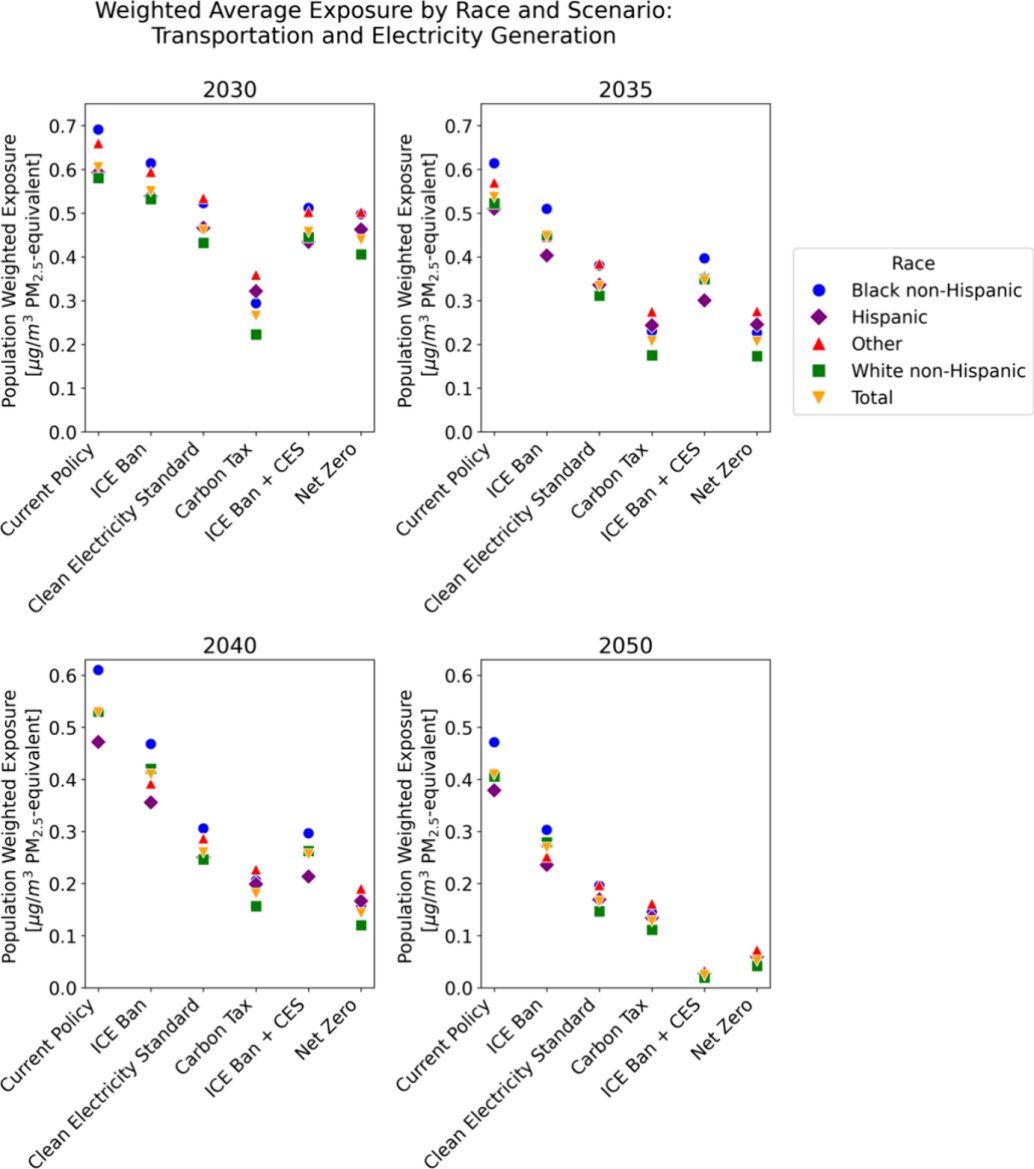
Population-weighted average PM_2.5_-equivalent exposure by race and scenario over time. Emissions are from both on-road transportation and EGUs.

While Figure 2 clearly demonstrates the overall trends in exposure and disparity for the different racial groups, the steep decline in emissions from 2020 to 2030 makes the comparison between scenarios in later years more difficult. Figure 3 shows weighted average PM_2.5_ exposure from on-road vehicles and EGUs by scenario and race in 2030, 2035, 2040, and 2050. The figure displays several key trends. First, by 2050, under net zero and a clean electricity standard combined with a ban on on-road vehicles, weighted average exposure from EGUs and on-road vehicles is near zero for all racial groups. Under the current policy, disparities remain. Despite emission reductions driven by the IRA and the falling costs of clean energy technologies, Black non-Hispanics have a higher population weighted-average exposure than all other racial groups in this baseline scenario: They are exposed to 13% higher PM_2.5_ concentrations from on-road vehicles and EGUs than the average American in 2050 barring additional policy measures. This is particularly notable because in the first time period, the average PM_2.5_-equivalent exposure for Black non-Hispanic Americans is equivalent to the total population average exposure, meaning that population-weighted exposure disparity is zero. Steep reductions in emissions from on-road vehicles primarily benefit Hispanic and Other groups (Figure 1), creating a disparity for Black non-Hispanic Americans, despite the fact that exposure decreases for all groups. This trend is true under other policies as well; in 2040, Black non-Hispanics are exposed to higher levels of PM_2.5_ equivalents than any other racial group in four of the six modeled policies.

Hispanic Americans have the lowest population-weighted average exposure in 2050 under the current policy, with exposures 8% lower than that of the average American. This is driven primarily by the high fraction of Hispanics living in California and other western states with aggressive clean energy and zero-emission vehicle targets.

When the policy targets only electricity emissions (CES), White non-Hispanic Americans have the lowest PM_2.5_ exposure of any racial group in every time period as they are disproportionately unaffected by on-road transportation emissions. This is not the case when the policy only targets transportation emissions (ICE ban); in that case, Hispanics have the lowest exposure by 2040. The CES leads to a 2040 U.S. population average exposure of 0.26 μg/m^3^ compared to 0.41 μg/m^3^ under the ICE ban. Figure 3 also demonstrates the importance of policy timing. The CES and vehicle bans do not start until 2030, while the carbon tax goes into effect in 2025. This leads to earlier reductions in net exposure and disparity. Despite early reductions, exposure under the carbon tax does not decrease as much in the later time periods relative to 2025 and 2030. The tax level is insufficiently high to spur large changes in emissions beyond what is achieved in the late 2030s, so concentrations stay approximately constant in the last four modeled time periods.

**Figure 4:**
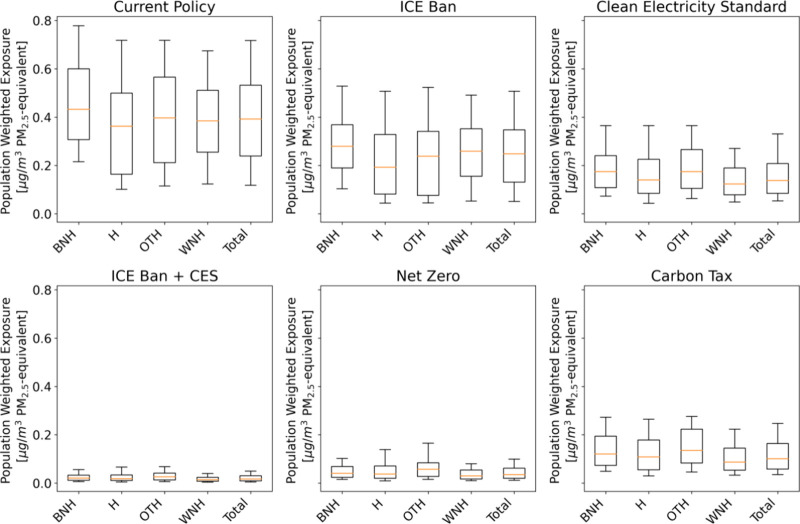
Population-weighted exposure distributions (25th percentile, median, and 50th percentile) across races and policy scenarios. The error bars represent the 10th and 90th percentile values. Values are reported only for 2050. “Total” displays trends for the U.S. population as a whole. In 2020, the modeled median exposures for Black non-Hispanics, White non-Hispanics, Hispanics, and other racial groups are 2.14, 1.76, 2.98, and 3.20, respectively.

Figure 4 illustrates the distribution of 2050 population-weighted average exposure. The current policy has the largest range between the highest and lowest exposures for all racial groups, indicating more heterogeneity across counties in the absence of climate policy. The median and population-weighted average exposure values displayed in Figures 2 and 3 do not capture the fact that there are clear winners and losers within racial groups, especially in the current policy. Black non-Hispanic Americans living in counties on the right-hand tail of the distribution end up exposed to concentrations 0.3 μg/m^3^ greater than those in counties exposed to the median level of air pollution. This primarily results from coal- and natural gas-fired power plants that remain online. These remaining point sources disproportionately affect a small number of counties and are particularly harmful for Black non-Hispanic Americans. The 90th percentile value for Black non-Hispanic Americans in the current policy is 0.12 μg/m^3^ higher than the 90th percentile for the population as a whole.

By 2050, the current policy and ICE ban have the highest remaining disparities. Under the ICE ban (current policy), the median exposure for Black non-Hispanics is 0.03 (0.04) μg/m^3^ higher than that for the population as a whole. For Hispanics, median exposure across all years under the ICE ban (current policy) is 0.06 (0.03) μg/m^3^ lower than the population as a whole. Figures 3 and 4 also demonstrate that the CES scenario leads to greater emission reduction than the ban on new ICE vehicles. This is driven by the turnover rate of existing vehicles and by a slight increase in electricity sector emissions under an ICE ban in the absence of stricter clean electricity policy.

### 3.2 Mortality

The discussion thus far has focused on disparities in the PM_2.5_-equivalent exposure. However, this may underestimate disparities in health outcomes.^81,83^ As discussed in Section 2.4.2, we estimate a range of deaths due to emissions from EGUs and transportation vehicles using different relative risks and baseline mortality rates by race. We calculate three distinct estimates for deaths for each racial group. All estimates are calculated across the full modeled time horizon (2020–2050). The first estimate assumes a constant relative risk and age-specific mortality rate, eliminating any variation in health outcomes by race. The second implements a constant relative risk but uses age- and race-specific mortality rates. The third assumes a race-specific relative risk and age- and race-specific mortality rates. There is still considerable uncertainty over how relative risk values may vary among demographic groups. Hence, we view this calculation as an illustrative exploration of how differential relative risks may influence future policy outcomes.

All scenarios reduce cumulative air pollution-related deaths from 2020 to 2050 relative to the current policy, as shown in Table 2. The carbon tax avoids the most deaths (164,700–236,800). Importantly, the net-zero and carbon tax scenarios would also avoid air pollution-related deaths from other sectors of the economy, as the policies apply system-wide, but we quantify deaths only from transportation and electricity generation. Despite the net-zero scenario reaching lower emissions by 2050, the carbon tax avoids more cumulative deaths, again highlighting the importance of near-term emissions reduction.

**Table 2:** Cumulative Avoided Deaths from 2020 to 2050 due to Air Pollution from On-Road Transportation and Electricity Generation

scenario	cumulative avoided deaths
ICE ban	40,900–62,700
clean electricity standard	92,000–133,300
ICE ban + CES	108,300–161,400
net-zero	135,500–197,300
carbon tax	164,700–236,800

The ICE ban avoids fewer deaths than any other scenario, partially resulting from increased electricity emissions in some years from electric vehicles without additional clean electricity policy. There are 65 unique U.S. counties where, in at least one modeled year, deaths are higher under the ICE ban than under the current policy due to increased emissions from electricity generation.

**Figure 5:**
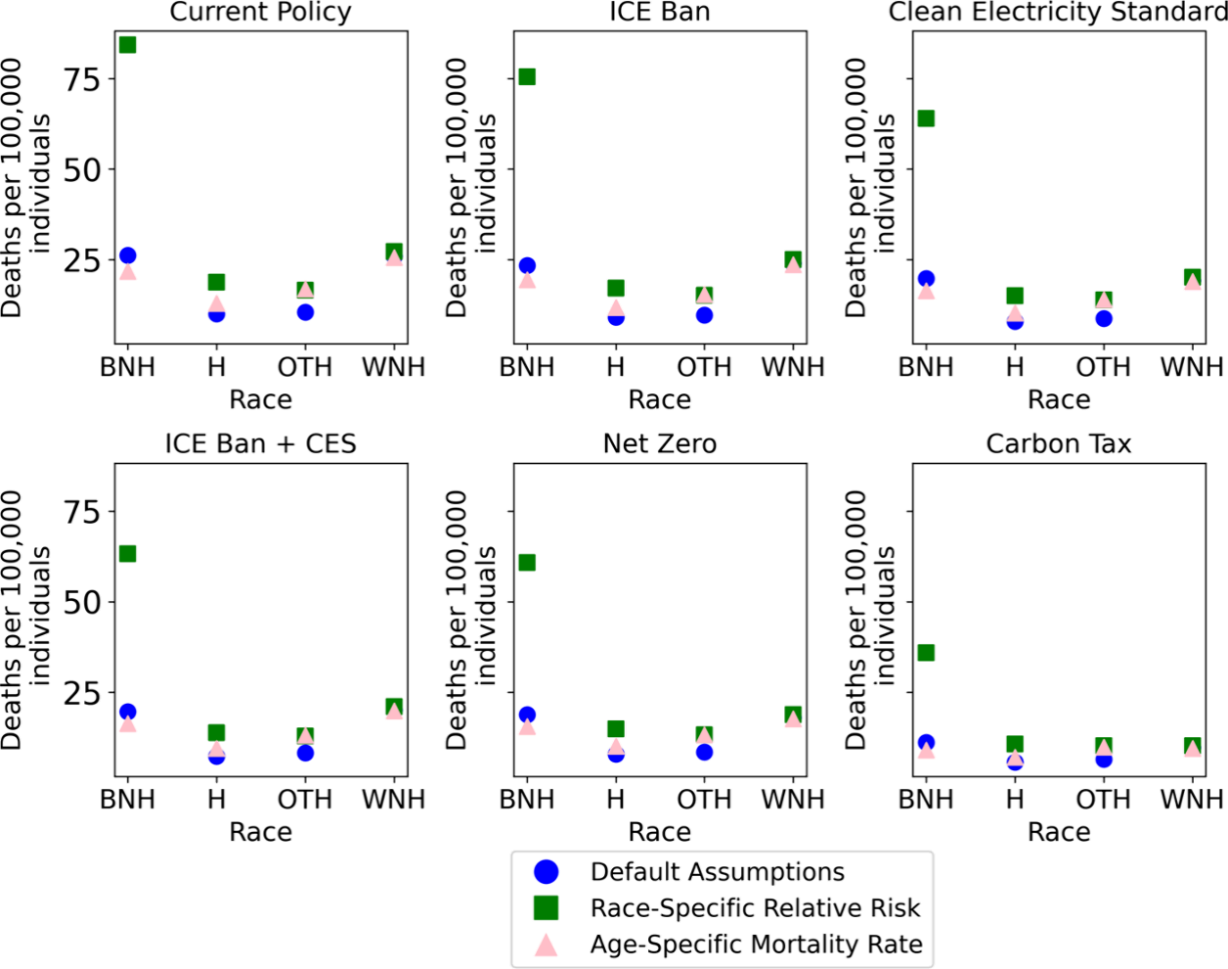
Deaths per 100,000 individuals by race and scenario in 2030. Default assumptions: constant relative risk and race- and age-specific mortality rate.

Cumulative avoided deaths illustrate the national impact of each policy. We also quantify per capita deaths by race. Figure 5 shows deaths per 100,000 individuals due to air pollution from on-road transportation and EGUs by race and scenario in 2030. The figure displays that for Black non-Hispanics per capita deaths change drastically when we use race-specific relative risks. We display results in 2030 as enough time has passed to highlight heterogeneity between the policy scenarios, but emissions remain high enough to emphasize the importance of relative risk selection. In all scenarios, race-specific relative risk for Black non-Hispanics leads to a 3-fold increase in estimated per capita deaths relative to the constant relative risk case. Institutionalized practices such as redlining, discriminatory neighborhood classification by mortgage lenders, and the placement of power plants and industrial facilities in economically disadvantaged neighborhoods with higher fractions of people of color have all resulted in non-White Americans being exposed to systematically higher levels of air pollution.^94,95^ Further, disadvantaged communities have less healthcare coverage and reduced access to healthcare, meaning that when these individuals do get sick, they are more susceptible to adverse outcomes.^94^

When examined alongside Figure S.1, Figure 5 also demonstrates the importance of age-specific mortality rates. Although White non-Hispanics have the lowest weighted-average exposure across all years in every scenario, this population does not always have the lowest per capita deaths. This can be attributed to the age distribution. White non-Hispanics are, on average, much older than other populations, and older individuals have higher mortality rates.

## 4 Discussion

This work adds to the growing discourse on equitable decarbonization pathways. Our results demonstrate that disparities between White non-Hispanics and other racial groups persist until at least 2040, even under aggressive decarbonization policies, although exposure and exposure disparity both decrease markedly over time. In the absence of climate policy, disparities exist, even in 2050. While we observe this trend under the current policy, disparity and exposure are lower under policies, such as a carbon tax, net-zero targets, an ICE ban, and a clean electricity standard. By 2050, only the ICE ban combined with a clean electricity standard completely eliminates exposure disparities. The current policy and ICE ban lead to the highest remaining disparities by 2050, especially for Black non-Hispanics, emphasizing the need for additional policy measures to address inequities. Policy scope plays a crucial role, as demonstrated by the difference in disparity outcomes between scenarios targeting only transportation emissions (ICE ban) or electricity emissions (CES) and those addressing both transportation and electricity emissions (ICE ban + CES and net-zero carbon tax). The timing of policy implementation also influences exposure outcomes, highlighting the importance of early emission reductions for achieving equity goals.

Our mortality risk analysis reveals the carbon tax as a particularly impactful strategy, avoiding the highest number of cumulative deaths. However, the ICE ban lags in avoided deaths due to increased electricity emissions in some years, supporting results found in other recent research.^96^ Per capita deaths by race reveal a nuanced picture of equity outcomes from decarbonization. Calculating mortalities with a race-specific relative risk results in substantially higher per capita deaths for Black non-Hispanics across all scenarios relative to all other racial groups, highlighting the importance of additional research to decrease uncertainty surrounding health risk by race.

Our results come with caveats due to parametric and structural uncertainty in each analysis step. Temoa’s modeling framework includes techno-economic parameters for technologies out to 2050. While we derive data from reputable sources such as government research laboratories and peer-reviewed literature, it is impossible to forecast these parameters exactly. The model structure also does not account for the real-world stakeholder heterogeneity in the energy system, the political landscape, and non-economic drivers of energy system decisions.^97^ In the downscaling algorithm, the population data set contains uncertainties about the precise demographic makeup of the U.S. Further, our model serves only as a simulation of possible future outcomes, not as a prediction. AP3's reduced-complexity dispersion and chemistry modeling approximates the fate and transport of emissions, and while the EPA directly monitors some emission sources, many NEI data observations are estimated.

Concentration–response also contributes to uncertainty, although we attempt to account for this by estimating mortalities with multiple relative risk and mortality rate estimates. Finally, our work does not consider any modeled policy’s cost burden distribution. While Temoa reports cost differences between the different scenarios, it does so at a regional level and from a system-planner perspective. Estimating downscaled cost impacts is beyond the scope of this analysis.

The spatial resolution of our analysis is limited to the county level by our downscaled data inputs. For example, we are unaware of any population projection data that are more granular than the county level. While it would theoretically be possible to use census-tract population estimates for the present-day population due to differing birth and death rates by race and projected immigration trends, it would not be reliable to assume that present-day census tract population estimates will hold out to 2050. Furthermore, the spatial surrogate that we use to downscale transportation emissions (EPA’s MOVES) is reported at the U.S. county level. These limitations of data necessarily mean that our analysis will miss near-source disparities. While these disparities are critical to understand, they are beyond the scope of this analysis and would be better assessed in a study with a more limited temporal and regional scope.

Despite model limitations, our results add to the literature, demonstrating the benefits of emissions beyond climate goals. By tying an ESOM to an IAM, we can estimate future equity outcomes resulting from the energy transition in multiple sectors, which are critical to ensuring an equitable transition to clean energy. If a particular policy reduces exposure but not disparity, then a policymaker could consider additional legislation preferentially targeting emission sources located in proximity to marginalized communities. Our modeling framework can serve as a guide for policymakers to achieve their equity-minded goals. When equity is a policy goal, it is necessary to consider the total exposure, exposure disparity across groups, and the distribution of outcomes within groups. While air pollution exposure is only one way to quantify equity outcomes, the direct connection between air pollution and increased mortality makes it an important one.

## Supporting Information Available

Supplemental figures; details on EGU downscaling; downscaling algorithm evaluation; air quality modeling; mortality risk and scenarios; marginal damages by effective height (PDF)
