# Supplementary material for: Climate Policy Reduces Racial Disparities in Air Pollution from Transportation and Power Generation
Source: Environ Sci Technol. 2024 Nov 26;58(49):21510–22. doi: 10.1021/acs.est.4c03719 (PMC11636253; doi:10.1021/acs.est.4c03719)
Supplement: Supplementary file 1 — es4c03719_si_001.pdf [file es4c03719_si_001.pdf]

Supplemental Information

Title: Climate Policy Reduces Racial Disparities in Air Pollution from Transportation and Power Generation

Authors: Katherine H Jordan[1*]; Luke R Dennin[1]; Peter J Adams[1,2]; Paulina Jaramillo[1]; & Nicholas Z Muller[1,3,4].

Affiliations: [1]Engineering and Public Policy, Carnegie Mellon University, 5000 Forbes Ave, Pittsburgh, PA, 15213, USA.; [2]Civil and Environmental Engineering, Carnegie Mellon University, 5000 Forbes Ave, Pittsburgh, PA, 15213, USA; [3]Tepper School of Business, Carnegie Mellon University, 5000 Forbes Ave, Pittsburgh, PA, 15213, USA; [4] National Bureau of Economic Research, 1050 Massachusetts Avenue, Cambridge, MA, 02138.

*Corresponding Author contact: khjordan@andrew.cmu.edu

Pages: 20
Figures: 7
Tables: 5

I. Supplemental Figures

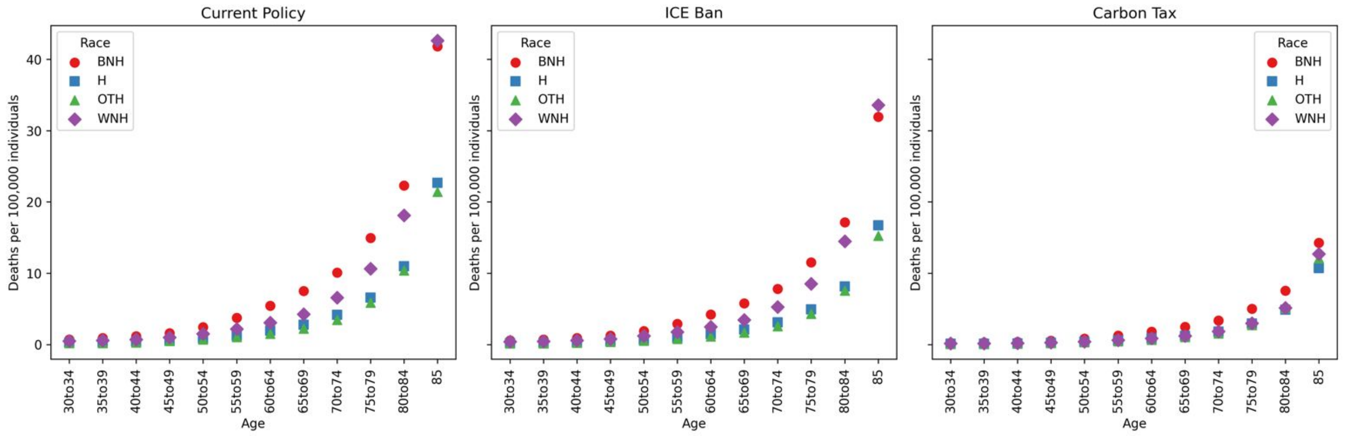

Figure S.1: Age- and race-specific deaths per 100,000 individuals for three select scenarios.

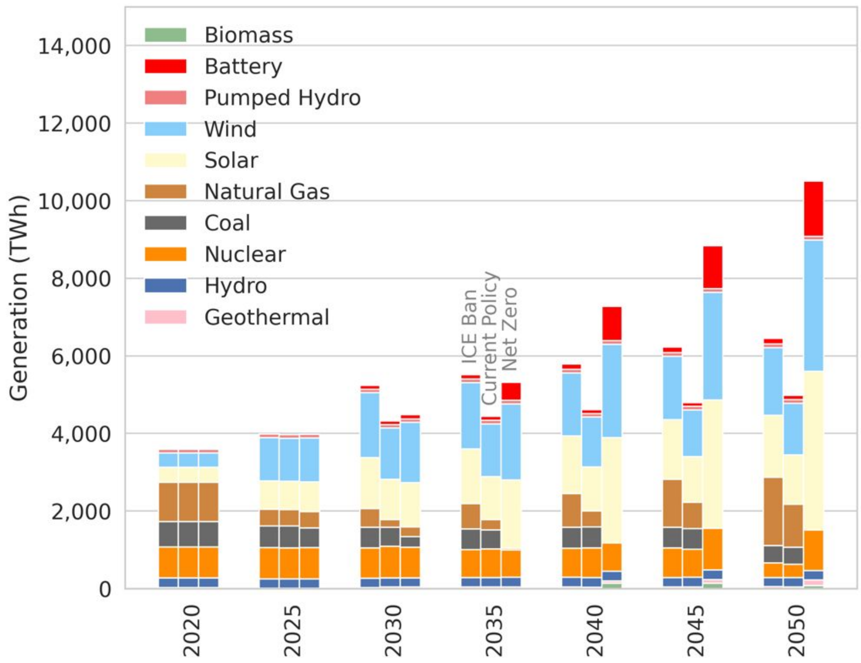

Figure S.2: Electricity generation by source under the ICE Ban, Current Policy, and Net Zero scenarios

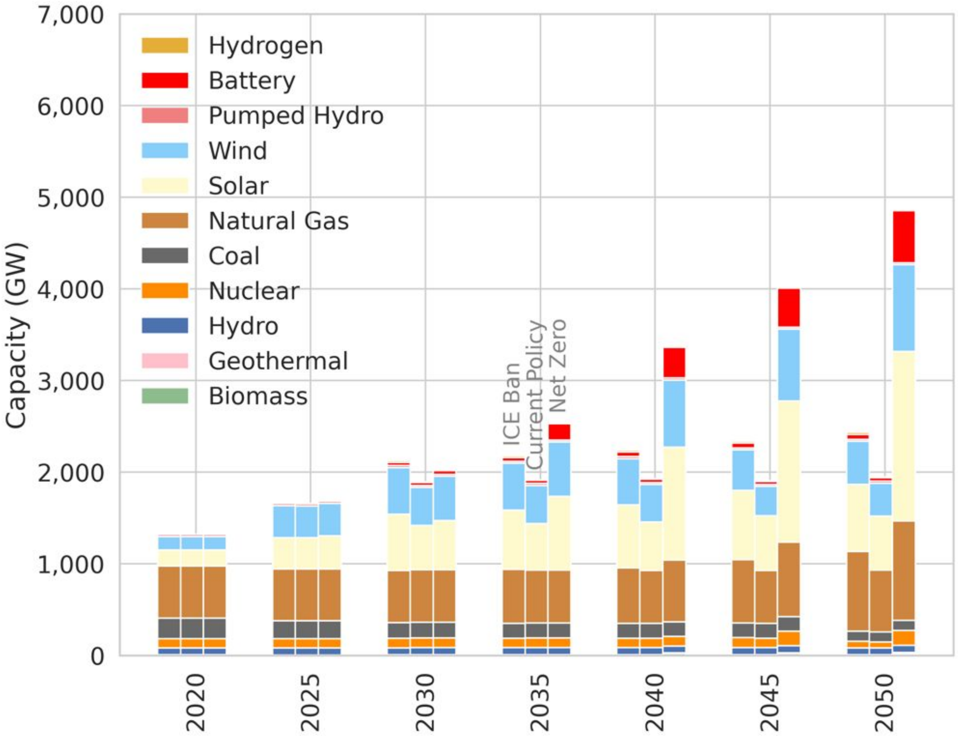

Figure S.3: Electric capacity by source under the ICE Ban, Current Policy, and Net Zero scenarios

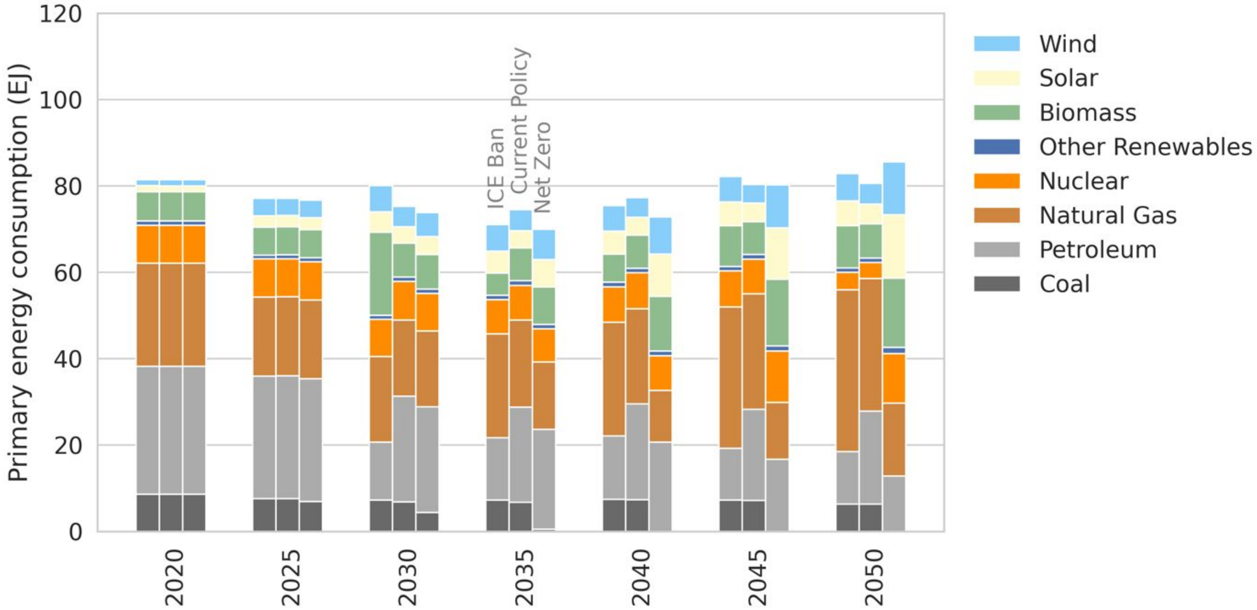

Figure S.4: Final energy consumption by source under the ICE Ban, Current Policy, and Net-Zero scenarios.

II. Details on EGU Downscaling

To determine the location of future capacity, we implement a grow-in-place heuristic. We use data on planned EGUs and EGUs that have retired since 2002 from December 2021's Form EIA-860M 'Monthly Update to Annual Electric Generator Report' to map existing and planned facilities to Temoa's capacity [1]. We use this dataset's geographical data, energy source code, and the nameplate capacity (MW) of plants. We then pull new fossil capacity (MW) from Temoa by fuel and cluster. To estimate the new capacity's location, we pull all planned EGUs in the cluster's region from Form EIA-860M, irrespective of plant type (combined cycle, combustion turbine, etc.). If the sum of the planned capacity in that region is greater than the new cluster's capacity from Temoa, we choose a random group of the planned plants whose capacities sum to between 0.75x and 1.25x the capacity required by the Temoa cluster. We assume the new capacity is built at the same sites as this list of planned sites. If planned capacity is insufficient, we repeat the process, adding in retired capacity from the region. If the sum of the planned and retired capacity in the region is less than the new cluster's capacity, we assign all of the planned and retired plants in that region to the new cluster. This implicitly assumes that some of the new plants will have a higher capacity than planned (if the plant is planned) or than it had when operating (if the plant is retired). We do not have data on average annual generation for the retired or planned plants, so we assume that generation scales with capacity in each cluster. That is, if a plant's capacity is equal to 5% of the cluster's total capacity, we assume the new plant generates 5% of the total electricity generated in that cluster.

III. Downscaling Algorithm Evaluation

Models with a multi-decadal time horizon cannot be validated [2]. However, to better understand the uncertainty present within the downscaling framework, we compare modeled county-level emissions from the transportation and electric sectors to existing data on emissions. This exercise should not be viewed as a direct comparison, but rather as a sanity check on the downscaling method. Temoa's first time period spans from 2020 to 2024. Even under the Current Policy scenario, Temoa simulates a changing energy landscape. For example, in 2022, US coal-fired powerplants generated 828 TWh of electricity [3]. Temoa simulates 654 TWh of coal-fired electricity generation in the first time period. These differences are unsurprising since Temoa assumes a unitary decision-maker and does not account for behavioral and market dynamics that occur in the real world.

In this exercise, we compare county-level NO_x emissions in the electric and transportation sectors from Temoa to the EPA's National Emissions Inventory (NEI) [4]. The EPA publishes the NEI approximately every three years. The most recent data comes from 2020, but due to effects from COVID-19, 2020 is likely not a representative year. As a result, we compare modeled downscaled emissions to both the 2020 and 2017 NEI. In the electricity sector, we also compare our results to the EPA's Emissions & Generation Resource Integrated Database (eGRID) [5]. We compare our results and NEI results to the most recent eGRID release, which contains data from 2021.

Figure S.4 compares Temoa’s simulated on-road transportation emissions to the NEI in 2017 and 2020, showing the absolute difference between the two datasets. We compare NEI results aggregated to the county-level to our downscaled simulation. In the figures, a positive value indicates that Temoa projected higher emissions than the NEI reported. We expected Temoa’s emissions projections to be lower than the 2017 projections due to increased vehicle electrification and improvements to vehicle efficiency. We anticipated that NEI 2020 emissions may be lower than Temoa’s simulated emissions due to effects from COVID-19. Figure S.4 shows strong agreement between the NEI and Temoa, with the distribution shifting slightly between the two NEI data years, in line with our hypotheses.

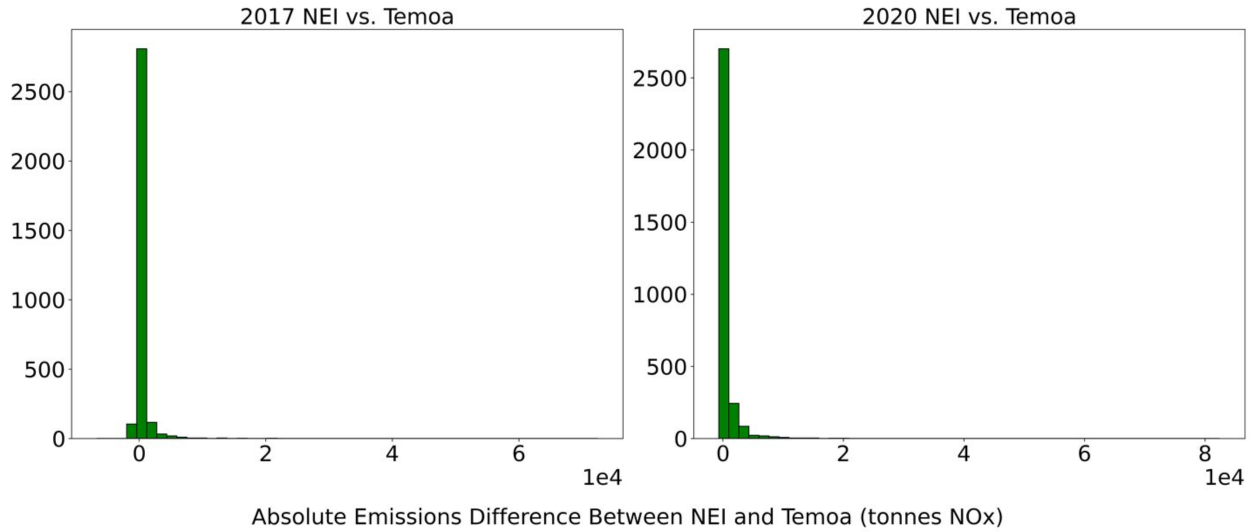

Figure S.5: Difference between NOx emissions reported by the National Emissions Inventory and emissions simulated by Temoa and downscaled to US counties. Emissions reported in metric tons. Left: 2017 NEI vs. Temoa. Right: 2020 NEI vs. Temoa.

Both histograms are centered near zero, indicating broad alignment with the NEI. The most significant outliers represent counties including Los Angeles and Maricopa counties, where total emissions are high, so a small percent difference appears large in absolute magnitude.

Next, we compare simulated NO_x emissions from electric power generation to reported emissions from the NEI and eGRID. The NEI and eGRID datasets required some data filtration. To select only EGUs from the NEI data, we filtered the column ‘naics_description’ to include only sources described as ‘Fossil Fuel Electric Power Generation’ or ‘Electric Power Generation.’ We also specified that ‘facility_source_type’ must be ‘Electricity Generation via Combustion.’ For the eGRID data, while all sources are powerplants, we filtered ‘Plant primary fuel category’ to include only ‘GAS’ and ‘COAL.’

We began by comparing the 2017 and 2020 NEI datasets to 2020 and 2021 eGRID to understand both interannual variability and variability between data sources (Figure S.4). This inter-dataset comparison reveals two trends. First, in counties where both datasets report emissions, there is general agreement across datasets and time periods. Second, there are many counties where either eGRID or NEI reports positive emissions, but not both. Figure S.5 illustrates this trend, showing the distribution of the percent difference between the different datasets. In order to avoid positive or negative infinities, we replace all 0 values with 1E-4. This

leads to large outliers, all of which are displayed in the figure. The top row of Figure S.4 shows the raw data and the bottom row shows a zoomed in version to highlight outliers.

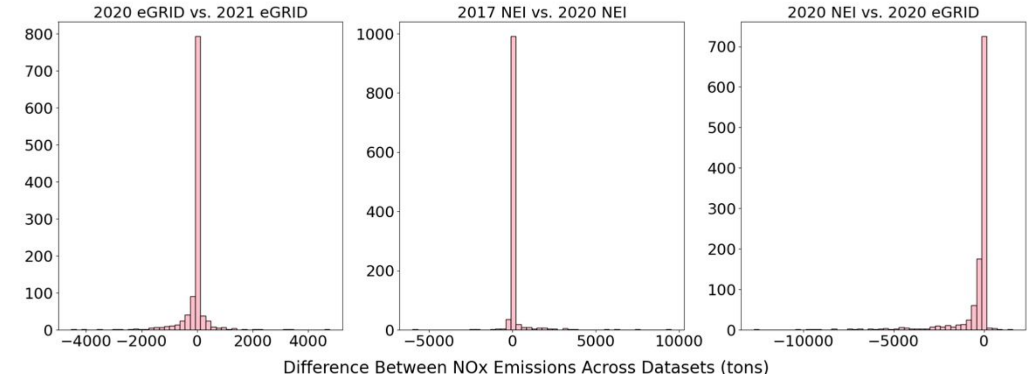

Figure S.6: Absolute emissions comparison between eGRID and the National Emissions Inventory [kt].

When both Temoa and NEI or eGRID report emissions, there is largely agreement, but there are many counties where one source (Temoa, NEI, eGRID) reports emissions and at least one other does not. The downscaled emissions from Temoa align most closely with the NEI, demonstrated in Figure S.6

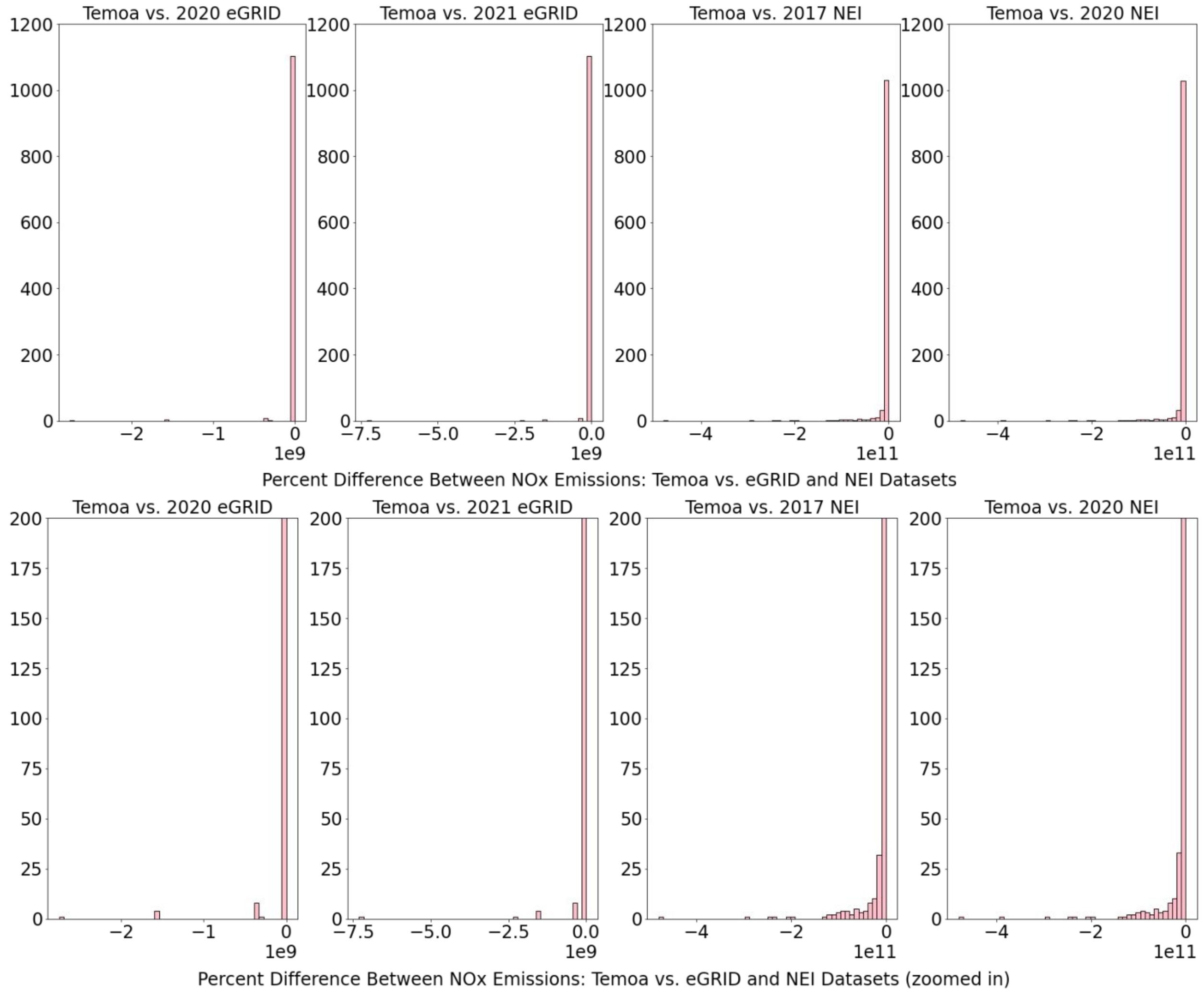

Figure S.7: Percent difference between NO_x emissions from power generation simulated by Temoa and reported by existing data source. Columns: (1) Temoa vs. 2020 eGRID (2) Temoa vs. 2021 eGRID (3) Temoa vs. 2017 NEI (4) Temoa vs. 2020 NEI. Rows: (1) Raw data (2) Zoomed in to more clearly display outliers.

IV. Air Quality Modeling

We use select modules of the AP3 integrated assessment model to connect emissions to $PM_{2.5}$ exposure and mortality risk for three criteria air pollutants: primary $PM_{2.5}$, SO_2, and NO_x. We summarize AP3 herein; however, more details can be found in Dennin & Muller (2024)'s supplementary information [6] as well as in Clay et al. (2019) [7] and Tschofen et al. (2019) [8]. AP3 [9] is the third iteration of the Air Pollution Emissions Experiments and Policy analysis (APEEP) model [10,11].AP3 uses all emissions of primary $PM_{2.5}$, SO_2, and NO_x and also NH_3 and VOCs, provided by the EPA's NEI, to estimate baseline concentrations of ambient $PM_{2.5}$ in every county in the contiguous US [12]. We use the 2017 NEI to compute a baseline against which we assess the $PM_{2.5}$ concentrations from marginal emissions (i.e., one additional short ton). This baseline accounts for ambient $PM_{2.5}$ levels resulting from nationwide emissions, including directly emitted $PM_{2.5}$, organic aerosols from VOCs, ammonium sulfate ($(NH_4)_2SO_4$) from NH_3 and SO_2, sulfate (SO_4^{2-}) from SO_2, and ammonium nitrate (NH_4NO_3) from NH_3 and NO_x. The

formation of each subspecies of $PM_{2.5}$ is dependent on the unique atmospheric dispersion and chemistry processes associated with the release of its source criteria air pollutant.

AP3 models ground-level sources differently than point sources. Moreover, the model treats point sources differently, a function of their effective heights (physical stack height plus the plume rise of released emissions) [13]. Effective heights are calculated following Turner (1994) [13]. Stack and discharge parameters are obtained from the Sparse Matrix Operator Kerner Emissions (SMOKE) flat files, provided by the Environmental Protection Agency (EPA) [14]. Weather parameters come from reanalysis data provided by the National Centers for Environmental Prediction (NCEP), with data sourced from the Physical Sciences Laboratory of the National Oceanic and Atmospheric Administration (NOAA) [15]. We compute the annual average of surface-level temperatures and horizontal wind speeds using daily average data, spatially resolved in a 2.5-degree latitude by 2.5-degree longitude grid. Each county is assigned the weather data from the grid cell in which it is located. If a county spans multiple grid cells, the data are averaged based on the area of each cell. Our methodology assumes stable conditions, an average lapse rate of -0.0065 K/m, and considers that, for each facility, the dominant rise mechanism is the one producing the greater rise between buoyant and momentum effects.

AP3 models three bins for point sources: low, medium, and tall [11]. For low and medium, emissions are modeled as being released from the population-weighted centroid of the county in which they are released. (Ground-level emissions are also assumed to be released at the counties' population-weighted centroids.):

- The low bin contains facilities with effective heights of less than 250 meters.
- The medium bin is designed for facilities with effective heights greater than 250 meters and less than 500 meters.
- When the APEEP) model was first developed [10], facilities with effective heights greater than 500 meters were uniquely modeled—making up the "tall stacks bin inventory."

The tall bin's emissions are modeled from the coordinates of the facility and the effective heights of its stacks. However, any new facility with an effective height greater than 500 meters is not modeled in the tall bin but is instead placed in the medium bin. For this study, all transportation emissions are modeled from the ground-level bin. All EGU emissions are modeled from the medium stacks bin. This methodology for EGU emissions allows us to work at the county level, the spatial resolution of our downscaled simulated emissions, and avoid matching (and potentially mismatching) simulated emissions to specific facilities in the tall bin.

It is important to understand the implications of this assumption and its limitations. Hence, we categorize the "existing fleet" of EGUs into bins based on available data. We analyze point source data provided by the NEI for facilities involved in power generation in 2017 [12]. This dataset is then filtered to include only facilities located in counties modeled by Temoa in 2020. Out of the 1,105 observed EGUs in the resulting dataset, 35% lack the necessary data to compute effective heights. These facilities do not have smokestack parameters reported in EPA's SMOKE flat files [14], which we can hypothesize corresponds with facilities that do not have tall smokestacks. This gives us justification to assign them to the low stacks bin of AP3. Still, given the uncertainty associated with this assumption, we exclude these facilities from the low stacks bin in Table S.1 (tracking them instead a *NA*), which summarizes the number of facilities by AP3 bin and sums total emissions for each.

Table S.1 shows that, in 2017, most effective heights among EGUs were below 500 meters (again, corresponding with AP3's tall bin), with only about 3% exceeding that height. However, the distribution of emissions tells a different story: no more than 10% of any pollutant was emitted from facilities with effective heights under 250 meters (corresponding with AP3's low bin). About one-third of facilities had effective heights between 250 and 500 meters (corresponding with AP3's medium bin), yet these accounted for roughly two-thirds of total emissions. Overall, most facilities do not have tall stacks, but most emissions are not from low stacks.

Table S.1: Electric generating units and NO_x, primary $PM_{2.5}$, and SO_2 emissions by effective height and corresponding AP3 bin. Note: facilities and emissions characterize 2017 and are from the National Emissions Inventory [12]. Smokestack parameters are from SMOKE flat files [14].

**Effective Height**	**Corresponding AP3 Bin**	**Electric Generating Units**		**Emissions**			**Percentage by Bin**		
				**NO_x**	**$PM_{2.5}$**	**SO_2**	**NO_x**	**$PM_{2.5}$**	**SO_2**
< 250 meters	Low	327	30%	56.1	6.32	53.4	9.1%	10%	6.5%
250-500 meters	Medium	361	33%	419	43.6	513	68%	71%	62%
> 500 meters	Tall	34	3.1%	141	11.0	258	23%	18%	31%
NA	Unknown	383	35%	1.96	0.650	0.154	0.3%	1.1%	0.0%

Notably, effective heights change over time because plume rise depends on varying parameters such as exit velocity, gas temperature, and atmospheric conditions [13]. As a result, some facilities with 2017 effective heights under 500 meters are still categorized in AP3's tall stacks bin inventory due to the model's original structure [10,11]. The 52 EGUs that are included in (1) the 1,105 observed EGUs in Table S.1 and (2) AP3's tall stacks bin inventory are evaluated in the top panel of Table S.2. These 52 EGUs are included in AP3's tall stacks bin because of taller effective heights that characterized the plants at previous points in time that may not still be applicable or were otherwise not applicable in 2017. We summarize these EGUs below:

- 42 EGUs, accounting for 72% to 73% of emissions from these 52 EGUs, have 2017 effective heights corresponding with the medium bin (250 to 500 meters)
- 4 EGUs, accounting for 10% to 12% of emissions from these 52 EGUs, have 2017 effective heights corresponding with the low bin (less than 250 meters)
- 6 EGUs, accounting for 16% to 19% of emissions from these 52 EGUs, have 2017 effective heights corresponding with the tall bin (more than 500 meters)

Conversely, several facilities with 2017 effective heights above 500 meters are not included in AP3's tall stacks bin inventory. This may be due to fluctuating effective heights over time or because newer facilities with heights over 500 meters have been defaulted to AP3's medium stacks bin. Of the 34 EGUs with 2017 effective heights exceeding 500 meters in Table S.1, 28 EGUs (82%) are not in AP3's tall stacks bin inventory and therefore are defaulted to the medium stacks bin. These EGUs and the others outside of AP3's tall stacks bin inventory are summarized

in the bottom panel of Table S.2. AP3's inflexible structure for tall stack (mostly EGU) facilities is a limitation of the model, which is currently being addressed in ongoing research developing its successor, AP4 [16].

Table S.2: Electric generating units within and outside of AP3's tall stacks bin inventory. Note: facilities with effective heights > 500 meters were modeled distinctly upon APEEP's development [10,11].

Tall Stacks Bin Inventory	Effective Height	Electric Generating Units		Emissions			Percentage by Bin		
				NO_x	$PM_{2.5}$	SO_2	NO_x	$PM_{2.5}$	SO_2
52 EGUs Within	Low: < 250 meters	4	0.4%	12.2	1.00	16.3	2.0%	1.6%	2.0%
	Medium: 250-500 meters	42	3.8%	110	6.95	118	18%	11%	14%
	Tall: > 500 meters	6	0.5%	24.3	1.30	24.1	3.9%	2.1%	2.9%
1,053 EGUs Outside	Low: < 250 meters	323	29%	43.9	5.32	37.1	7.1%	8.6%	4.5%
	Medium: 250-500 meters	319	29%	310	36.6	395	50%	59%	48%
	Tall: > 500 meters	28	2.5%	116	9.71	234	19%	16%	28%
	NA	383	35%	1.96	0.650	0.154	0.3%	1.1%	0.0%

The takeaway is that bin assignment uncertainty and limitations exist whether or not we specifically distribute facilities to different bins by effective heights. Since our Temoa modeling does not operate at the facility level, assigning all EGU emissions to the medium stacks bin is the most logical approach. This bin is the best representative for EGUs given the limited facility-specific information available. Moreover, even if we attempted to allocate emissions from EGUs with effective heights over 500 meters to AP3's tall stacks bin, many would still default to the medium stacks bin (Table S.2's bottom panel). Additionally, many facilities that could be assigned to the tall stacks bin are better suited for the medium stacks bin based on 2017 effective heights (Table S.2's top panel). The primary potential for missing effective height differentiation lies with the low stacks bin, but these facilities contribute significantly fewer emissions than those with effective heights greater than 250 meters, making facility-specific distribution a persistent challenge.

AP3 uses source-receptor (S-R) matrices to model pollution concentrations in every receptor contiguous US county from all sources of emissions. These matrices are built using Gaussian plume mathematics, which characterizes three-dimensional atmospheric dispersion from the point of release [17–19]. AP3 acts as a representation of annually averaged atmospheric conditions; see Turner (1994) for a further explanation [20].

The following equations depict the air quality modeling of AP3. First, as shown in Equation S.1, emissions (e) from each source (S) are loaded in as vectors (E) by pollutant (p) and bin height (h). The S-R matrices, shown in Equation S.2, model the transport (t) of a short ton of pollution from each source to each receptor (R), again by pollutant and bin height. This transport is specific for each pollutant, accounting for relevant chemistry and deposition processes. Multiplying the emissions vectors by their associated S-R matrices, shown in Equation S.3, provides speciated concentrations (C) of pollution, by pollutant and bin height, in each contiguous US county resulting from all sources' emissions. The concentrations are then added

across the different bin heights for total speciated ambient pollution in every county resulting from emissions of NH_3, NO_x, primary $PM_{2.5}$, SO_2, and VOCs.

$$E_{p,h} = \begin{bmatrix} e_1 \\ \vdots \\ e_S \end{bmatrix} \quad \text{(Equation S.1)}$$

$$SR_{p,h} = \begin{bmatrix} t_{1,1} & \cdots & t_{1,R} \\ \vdots & \ddots & \vdots \\ t_{S,1} & \cdots & t_{S,R} \end{bmatrix} \quad \text{(Equation S.2)}$$

$$C_{p,h} = (E_{p,h})^T \times (SR_{p,h}) \quad \text{(Equation S.3)}$$

The formation of $(NH_4)_2SO_4$, SO_4^{2-}, and NH_4NO_3 depends on the equilibrium between total (gaseous plus particulate) ammonia, particulate sulfate, and total nitrate.[1] Specifically, AP3 models the interpollutant chemistry between ambient ammonia (ambient NH_3), sulfuric acid (H_2SO_4), and nitric acid (HNO_3) as they form particulates [21–23]. AP3 then aggregates all subspecies of ambient $PM_{2.5}$ to determine total concentrations in each county.

Ambient NH_3 reacts preferentially with H_2SO_4 to form $(NH_4)_2SO_4$. Any remaining, or free, ambient NH_3 can then react with HNO_3 to form NH_4NO_3.[2] Two regimes affect this formation: (1) nitrate-limited, where ambient NH_3 is in surplus, and (2) ammonium-limited, where HNO_3 is in surplus. The efficiency with which marginal emissions of NH_3 and NO_x form NH_4NO_3 depends on the regime in which a receptor county resides. Unlike emissions of NH_3 and NO_x, emissions of SO_2 will always contribute to ambient $PM_{2.5}$ formation, regardless of the availability of ambient NH_3, because SO_4^{2-} is always in the particulate phase.

A critical caveat for this study is that we only use marginal $PM_{2.5}$ concentrations considering a 2017 baseline. In other words, we assess simulated future emissions in their respective future years, but the marginal $PM_{2.5}$ concentrations per short ton of future emissions are those modeled in AP3 for 2017. Our use of a constant 2017 pollution baseline is based on data availability. The NEI is released every three years, which determines the years that can be modeled using the APEEP model (e.g., AP3 can model 2008, 2011, 2014, and 2017) [6,8,25]. Although the 2020 NEI was released last year [26], the development of this version of AP3 occurred before that release. Even if we were to use the 2020 NEI (or the 2023 NEI, expected to be released in 2026), concerns about changes in atmospheric chemistry profiles through 2050 would remain. The key issue is the potential for substantial changes in the relative concentrations of one or more pollutants between 2017 and 2050, which could significantly impact atmospheric chemistry in the U.S. For the sake of our methods (i.e., a constant 2017 pollution baseline to which marginal emissions contribute), it is ideal for the relative concentrations of the pollutants from emissions of SO_2, NO_x, and NH_3 to remain consistent over time. That said, these relative concentrations are likely to change in the future. For example, the policies evaluated in our study, which focus on heavy sources of SO_2 and NO_x emissions, would contribute to this change, ceteris paribus.

Evidence suggests that we should anticipate a future with abundant ambient NH_3 relative to H_2SO_4 and HNO_3. Since the 2000s, SO_2 and NO_x emissions in the U.S. have declined, while NH_3 emissions have remained steady [27]. These trends are mostly expected to persist through

---

[1]

[2] SO_2 and NO_x form H_2SO_4 and HNO_3 when they react with oxygen (O_2) and water (H_2O). For example, $2SO_2 + O_2 + 2H_2O \rightarrow 2H_2SO_4$. For more information on the atmospheric behavior of the family of NO_x compounds, see [24].

mid-century under the Representative Concentration Pathway (RCP) 4.5 scenario, although NH_3 emissions are likely to increase [28–30]. These contrasting trends are driven by expectations for key sectors. Tschofen et al. (2019) identified that SO_2, NO_x, and NH_3 damages were greatest from the utilities, transportation, and agriculture sectors, respectively [8]. While emissions reductions from the power sector and transportation are projected to continue [31,32], research suggests that NH_3 emissions will rise due to higher temperatures from climate change [33] and increased agricultural activity to support a growing population [34]. However, there are existing methods to reduce NH_3 emissions from agriculture [35], and Gu et al. (2021) found that NH_3 abatement is relatively cost-effective compared to NO_x abatement [36].

These expected changes are significant when considering the marginal concentrations (μ g/m^3) we anticipate from SO_2 and NO_x emissions (and NH_3, though it is not the focus of this study). Below is a summary of the key points:

- In the future, we expect reductions in SO_2 and NO_x emissions and increases in NH_3 emissions [28]. This will alter the relative concentrations influencing the ammonium, sulfate, and nitrate balance.
- As a result, SO_2 and NO_x emissions are likely to become more damaging on the margin due to the relative surplus of ambient NH_3.
- Pollution from SO_2 emissions will contribute to ambient $PM_{2.5}$ regardless of the presence of ambient NH_3. Without ambient NH_3, SO_2 will form particulate H_2SO_4 at a minimum [23]. However, with higher NH_3 emissions, the formation of $(NH_4)_2SO_4$ becomes more likely.[3]
- In contrast, NO_x emissions will not contribute to ambient $PM_{2.5}$ without free ambient NH_3.[4] As NH_3 emissions increase, HNO_3 is more likely to form NH_4NO_3, which contributes to ambient $PM_{2.5}$.

In conclusion, SO_2 and NO_x emissions are more damaging per ton on the margin in an NH_3-saturated environment compared to an NH_3-limited environment.

Hernandez (2023), which examined the sensitivity of health damages to changes in atmospheric chemistry, provides relevant insights[28]. Using a chemical transport model, the study projected that marginal annual damages from ambient $PM_{2.5}$ associated with SO_2 and NO_x emissions are expected to increase by 16% and 17%, respectively, from 2005 to 2055 when accounting only for interactions between sulfate, nitrate, and ammonium PM2.5 components. These are relatively modest changes in marginal damages, supporting the use of RCMs even for analyses decades in the future with different emissions baselines. Additionally, since our focus is on changes from 2017 to 2055, we need to account for the fact that over a decade of emissions reductions from SO_2 and NO_x already influences AP3's baseline. This period includes the most

[3] $(NH_4)_2SO_4$ is more damaging than H_2SO_4 when derived from the same quantity of SO_2 emissions. This is best illustrated by comparing their molecular weights: 132 g/mol for $(NH_4)_2SO_4$ and 98 g/mol for H_2SO_4.

[4] AP3 assumes that all NO_x emissions contribute to ambient $PM_{2.5}$, even in the absence of free ambient NH_3 (based on the modeled reaction between H_2SO_4 and NH_3). Since AP3 models annual average concentrations, it would be inaccurate to assume that no ambient $PM_{2.5}$ from NO_x would occur without modeled free NH_3. For instance, there may be periods during the year with high levels of ambient NH_3 and low levels of H_2SO_4, contrary to more typical conditions. On such days, HNO_3 could react with the available NH_3 to form NH_4NO_3. The statistics used in AP3 for this calculation are derived from calibration efforts involving the Comprehensive Air Quality Model with extensions (CAMx) model [37,38].

significant decrease in SO_2 and NO_x emissions relative to future years[28]. By 2017, AP3's baseline indicated that most counties had some level of free ambient NH_3, based on annual average concentrations. Therefore, by 2017, SO_2 was likely already forming more damaging $(NH_4)_2SO_4$. The formation of NH_4NO_3 from NO_x would vary by county depending on the extent of the availability of free NH_3. As a result, the expected increases in damages from SO_2 and NO_x emissions from 2017 to 2055 (rather than from 2005 to 2055) are likely to be less than the projected 16% and 17% increases.

Importantly, Hernandez (2023) also examined the extent to which NO_x contributes to tropospheric ozone in 2005 versus 2055 [28]. Ozone is a significant oxidant for VOCs, which form ambient $PM_{2.5}$ as secondary organic aerosols (SOAs). The study found that the marginal damages from NO_x emissions related to ozone formation are expected to increase by more than 500% from 2005 to 2055. This represents a limitation of the AP3 model and our study, as they do not account for the ambient $PM_{2.5}$ impacts of ozone on SOA formation or the health impacts of tropospheric ozone itself [39,40]. On the whole, Hernandez found that NO_x damages (including both organic and inorganic sources) would approximately double from 2005 to 2055.

Conversely, our analysis does not factor in the benefits associated with reduced ambient $PM_{2.5}$ from marginal NH_3 emissions due to fewer SO_2 and NO_x emissions. These co-benefits will accrue because ambient NH_3 will not contribute to $PM_{2.5}$ formation without H_2SO_4 or HNO_3 to react with. In fact, marginal damages from NH_3 emissions are projected to decline by 67% from 2005 to 2055 [28].

In summary, assuming that ambient NH_3 remains as relatively abundant as it was in 2017 is a straightforward and reasonable approach given the discussion above. The marginal ambient $PM_{2.5}$ concentrations from SO_2 emissions are likely to be associated with $(NH_4)_2SO_4$, both in 2017 and in the future. However, we can assert with confidence that these will not increase by more than 16% by mid-century. For NO_x emissions, the impacts vary by receptor location depending on the availability of free ambient NH_3, but we can reasonably assume that the net damages may double by mid-century. While adjusting our baseline for future assessments could provide more precise estimates, it would introduce significant complexities and potential distortions in our results.

We again note that our work is comparative in nature. As with our 2017 concentrations, we can assume that any errors in our future projections will be uniformly distributed (e.g., if NO_x becomes more damaging, it will be more damaging "everywhere"). The primary concern is whether there is a systematic link between agricultural activity (and NH_3 emissions) and (1) changes in emissions from electric generating units and transportation or (2) the locations of the subpopulations assessed in our study. We leave such investigations and further efforts to project atmospheric chemistry conditions to future research.

AP3 is calibrated using EPA Air Quality System (AQS) monitoring data [41]. Key statistics are the mean fractional error (MFE) and mean fractional bias (MFB), defined below—Equations S.4 and S.5, respectively. Model predictions of ambient concentrations ($C_{m,i}$) are compared to observed levels of ambient concentrations ($C_{o,i}$) for receptor county locations with AQS monitoring data (i):

$$MFE = \frac{1}{n}\sum_{i=1}^{n} \frac{|c_{m,i} - c_{o,i}|}{\left(\frac{c_{m,i} + c_{o,i}}{2}\right)} \quad (Equation\ S.4)$$

$$MFB = \frac{1}{n}\sum_{i=1}^{n} \frac{c_{m,i} - c_{o,i}}{\left(\frac{c_{m,i} + c_{o,i}}{2}\right)} \quad (Equation\ S.5)$$

AP3 is calibrated using the observed data. The procedure compares AP3-modeled ambient $PM_{2.5}$ concentrations to those measured at monitors and conducts several calibration steps to improve the prediction-observation fit. The primary calibration step is an iterative approach that reduces the MFE and the MFB by adjusting calibration coefficients applied alongside the source-receptor matrices. A secondary calibration step is also taken to adjust the share of ambient $PM_{2.5}$ from primary $PM_{2.5}$ emissions for the 2.5th percentile of counties with the greatest absolute difference between modeled and monitored pollution. Subsequently, in a tertiary calibration step, neighboring counties surrounding the secondary calibration-adjusted counties are also considered for adjustment, where monitoring data and modeling estimates support such decisions.

Table S.3: Performance metrics for AP3 following calibration. Note: monitored data are from the Environmental Protection Agency's Air Quality System [41]*. r = Pearson's correlation coefficient. n = number of AQS-monitored counties for each pollutant. Calibration efforts are from* [42,43].

**Year**	**Ambient Pollutant**	**Performance Metrics**			
		**MFE**	**MFB**	**r**	**n**
2017	**Total $PM_{2.5}$**	**0.292**	**0.012**	**0.523**	**603**
	Sulfate	0.329	-0.105	0.699	255
	Nitrate	0.492	0.070	0.624	251
	Organic Aerosols	0.398	-0.043	0.450	247
	Ammonium	0.996	0.995	0.546	132

These calibration efforts were conducted as part of the 2017 NEI update to AP3. We attribute the calibration work to Tschofen et al. (2023) [42] and Tschofen (2023) [43]. Furthermore, the calibration procedure built upon previous work [25,37,44]. Table S.3 summarizes the performance metrics for AP3 following calibration. AP3 performs within the performance standards set by the literature and summarized below [45,46]:

Boylan & Russell (2006): According to [45], a MFE ≤ +50% and a MFB ≤ ±30% suggests that a model has met its performance goal. [45] also notes that an MFE ≤ +75% and an MFB ≤ ±60% are acceptable for modeling and that minor species should have less stringent requirements. The Intervention Model for Air Pollution (InMAP) model [47] also considers this performance standard.

- Morris et al. (2005): According to [46], an MFE ≤ +35% and an MFB ≤ ±15% suggests that a model achieves "excellent" performance. [46] also classified an MFE ≤ +50% and an MFB ≤ ±30% (i.e., the main standard from [45]) as "good" performance. The Estimating Air pollution Social Impact Using Regression (EASIUR) model [48] also considers this performance standard.

For total $PM_{2.5}$ and sulfate, AP3 meets the "excellent" performance standard from Morris et al. (2005) [46]. Nitrate and organic aerosols meet the standard from Boylan & Russell (2006) [45]. Ammonium is the one subspecies that has a lower performance. We again emphasize that [45]

noted that minor species should have less stringent requirements and that ammonia is not a focus of this study.

AP3 builds out a pollution baseline, but its outputs are the marginal impacts of emissions—e.g., additional $PM_{2.5}$ concentrations in downwind locations from a small increase in emissions from a particular source. In other words, once the baseline concentrations are determined by modeling all emissions [12], policy analyses are conducted by running emissions experiments as outlined in the following steps:

1. Set the model to its baseline (i.e., $PM_{2.5}$ in every county from all emissions).
2. Add one short ton of emissions for one pollutant from an individual source.
3. Compute the marginal concentrations in every receptor county by subtracting baseline concentrations from the new concentrations with the marginal short ton added.
4. Reset the model to its baseline.
5. Repeat 2-4 for each source and pollutant combination.

Marginal concentrations of $PM_{2.5}$ are the output of AP3's air quality modeling for this study. Then, we multiply total (avoided) emissions by their associated marginal concentrations for total (avoided) $PM_{2.5}$ concentrations in every receptor county. This is an approach based on that conducted in previous work—e.g., [6,8,25,49].

V. Mortality Risk and Scenarios

We use a dose-response function from the epidemiological literature, shown in Equation S.6, to associate exposure to ambient $PM_{2.5}$ with premature mortality risk. The dose-response function models the expected change in mortality rates across populations from a change in $PM_{2.5}$. The inputs to the function are a β coefficient, the baseline mortality rate of the exposed population (y_0), and the change in ambient $PM_{2.5}$ pollution (ΔPM). β is further defined in Equation S.7. β is the natural log of the relative risk (RR) with a change in $PM_{2.5}$. Multiplying the expected change in mortality rate (Δy) by the corresponding population, depicted in Equation S.8, yields the premature mortality associated with the change in $PM_{2.5}$.

$$\Delta y = y_0\left(1 - \frac{1}{exp(\beta \times \Delta PM)}\right) \tag{S.6}$$

$$\beta = \frac{ln(RR)}{\Delta PM} \tag{S.7}$$

$$\Delta Mortality = \Delta y \times Population \tag{S.8}$$

Table S.4 shows the mortality scenarios used in this study. Krewski et al. (2009) reported information for populations of 30 or older [50]. The study reported relative risk (i.e., the chances of an event occurring in an exposed group vs. the chances of it happening in a control group) of all-cause mortality associated with a 10 μg/m^3 increase in ambient $PM_{2.5}$ exposure. This relative risk is used in Scenario 1 of Table S.4.

Table S.4: Input parameters for concentration-response function. Note: relative risk and baseline mortality are held constant across model time periods due to data availability.

	Relative risk [source]	Baseline mortality
Scenario 1	1.06 [ACS: Krewski et al. (2009)]	Changes with race-ethnicity and age
Scenario 2	Black non-Hispanic: 1.208<br>White non-Hispanic: 1.063<br>Hispanic: 1.116<br>Other: 1.096<br>[Di et al. (2017)]	Changes with race-ethnicity and age
Scenario 3	1.06 [ACS: Krewski et al. (2009)]	Changes with age

Di et al. (2017) reported information for populations of 65 or older [51]. The study also reported relative risk of all-cause mortality associated with a 10 μg/m^3 increase in ambient $PM_{2.5}$ exposure. However, Di et al. (2017) also reported relative risk differentiated by race/ethnicity subpopulation. As discussed in the manuscript, we assume these differentiated relative risks to apply not only to the studied population (i.e., 65 or older) but also to other adults found to experience damage by Krewski et al. (2009) (i.e., 30 or older). We assert that this methodology is not informed by the epidemiological literature but rather by the reasonable assumption that disparities existing for older age intervals may also exist for younger age intervals. These relative risks are used in Scenario 2 of Table S.4.

County-level mortality rate data are derived using population and mortality data from the CDC's WONDER [52,53]. Data are reported by county, age group, and, optionally, race/ethnicity. Where data are not available to compute mortality rates, first the state average, then the US Health and Human Services region average, and then the national average mortality rates are substituted in, as necessary. Table S.4's Scenario 1 and Scenario 2 consider mortality rates by county, age group, and race/ethnicity. Table S.4's Scenario 3 considers mortality rates by only county and age group.

A critical caveat for this study is that we only use mortality rates in 2017. Since our analysis extends far into the future, we miss potentially notable changes in baseline mortality rates. These could be related to various trends (e.g., changes in the health care system). Future work could further incorporate expected future mortality rates into prospective analyses such as that conducted herein.

VI. Marginal Damages by Effective Height

Because we model all EGUs from the medium stacks bin of AP3 (250 meters < effective height < 500 meters), it is important to explore the differences in impacts from various bin heights and consider how our results might change when using the low stacks or tall stacks bins instead of our default medium stacks procedure. Table S. presents emissions-weighted marginal damages (in deaths per short ton) for NO_x, primary $PM_{2.5}$, and SO_2 from all sources of pollution across the contiguous U.S. in 2017, as reported by the NEI [12]. The concentration-response

function uses relative risk from Krewski et al. (2009) [50] and all-person population counts and baseline mortality rates, both differentiated by age, provided by the CDC [52,53].

Table S.5: Marginal damages (in deaths per short ton) by AP3 bin. Note: marginal damages are emissions-weighted from all sources of pollution across the contiguous U.S. in 2017 as reported by the National Emissions Inventory [12].

Variable	AP3 Bin	Pollutant		
		NO_x	$PM_{2.5}$	SO_2
Marginal Damages (Deaths/Short Ton)	Low	1.89E-03	1.54E-02	7.44E-03
	Medium	1.05E-03	8.77E-03	4.29E-03
	Tall	9.55E-04	5.00E-03	4.11E-03
Versus Medium	Low	+80%	+76%	+73%
	Tall	-9.0%	-43%	-4.2%

Facilities in AP3's low bin have significantly higher marginal damages on average compared to the medium bin. Specifically, deaths per ton of NO_x, primary $PM_{2.5}$, and SO_2 are 81%, 76%, and 73% greater, respectively. Conversely, facilities in the tall bin have lower marginal damages on average than those in the medium stacks bin, with deaths per ton of NO_x, primary $PM_{2.5}$, and SO_2 being 9%, 43%, and 4% lower, respectively. Marginal damages from taller facilities are typically lower because their smokestacks are designed to disperse concentrated pollution away from ground-level populations, whereas lower facilities tend to have the opposite effect.

While Table S.5 demonstrates that low or tall facilities/emissions attributed to the medium bin could be notably mischaracterized, we again highlight that the goal of our paper is comparative in nature. Our focus is on how air quality changes for different populations under different pathways. In short, where we error, we error the same for all pathways. While the marginal concentrations and marginal damages from EGUs with lower effective heights may be higher and from EGUs with higher effective heights may be lower, we can assume that they will do so uniformly or randomly. We note that this is not a strictly valid assumption if there is a correlation between low or tall facilities/emissions and (1) where EGU emissions change (relative to other EGU emissions) or (2) where the subpopulations assessed herein live. However, we leave that investigation as well as any further efforts to evaluate our atmospheric transport assumptions to future work.

Supplemental Information References

(1) US Energy Information Administration. *Preliminary Monthly Electric Generator Inventory (based on Form EIA-860M as a supplement to Form EIA-860)*. https://www.eia.gov/electricity/data/eia860m/index.php (accessed 2022-03-17).

(2) DeCarolis, J.; Daly, H.; Dodds, P.; Keppo, I.; Li, F.; McDowall, W.; Pye, S.; Strachan, N.; Trutnevyte, E.; Usher, W.; Winning, M.; Yeh, S.; Zeyringer, M. Formalizing Best Practice

for Energy System Optimization Modelling. *Applied Energy* **2017**, *194*, 184–198. https://doi.org/10.1016/j.apenergy.2017.03.001.

(3) US Energy Information Administration. *Frequently Asked Questions (FAQs)*. https://www.eia.gov/tools/faqs/faq.php (accessed 2023-03-06).

(4) US Environmental Protection Agency's Office of Air and Radiation (OAR). *2017 National Emissions Inventory (NEI) Data*; Other Policies and Guidance; Air Emissions Inventories, 2017. https://www.epa.gov/air-emissions-inventories/2017-national-emissions-inventory-nei-data (accessed 2022-05-09).

(5) US Environmental Protection Agency's Office of Air and Radiation (OAR). *Emissions & Generation Resource Integrated Database (eGRID)*. https://www.epa.gov/egrid (accessed 2023-03-06).

(6) Dennin, L. R.; Muller, N. Z. Funding a Just Transition Away from Coal in the U.S. Considering Avoided Damage from Air Pollution. *Manuscript Submitted for Publication* **2024**.

(7) Clay, K.; Jha, A.; Muller, N.; Walsh, R. External Costs of Transporting Petroleum Products: Evidence from Shipments of Crude Oil from North Dakota by Pipelines and Rail. *The Energy Journal* **2019**, *40* (1), 55–73. https://doi.org/10.5547/01956574.40.1.kcla.

(8) Tschofen, P.; Azevedo, I. L.; Muller, N. Z. Fine Particulate Matter Damages and Value Added in the US Economy. *Proc Natl Acad Sci USA* **2019**, *116* (40), 19857–19862. https://doi.org/10.1073/pnas.1905030116.

(9) Muller, N. Z. *AP4 (AP3, AP2, APEEP) Model*. Nick Muller's Home Page. https://nickmuller.tepper.cmu.edu/APModel.aspx (accessed 2024-03-23).

(10) Muller, N. Z.; Mendelsohn, R. Measuring the Damages of Air Pollution in the United States. *Journal of Environmental Economics and Management* **2007**, *54* (1), 1–14. https://doi.org/10.1016/j.jeem.2006.12.002.

(11) Muller, N.; Mendelsohn, R. The Air Pollution Emission Experiments and Policy Analysis Model (APEEP) Technical Appendix. **2014**.

(12) U.S. EPA's Office of Air and Radiation. *2017 National Emissions Inventory (NEI) Data*. Air Emissions Inventories. https://www.epa.gov/air-emissions-inventories/2017-national-emissions-inventory-nei-data (accessed 2022-05-09).

(13) Turner, D. B. 3: Effective Height of Emission. In *Workbook of Atmospheric Dispersion Estimates: An Introduction to Dispersion Modeling*; CRC Press, Inc.: Boca Raton, FL, 1994; p 3.1-3.8.

(14) U.S. EPA's Office of Air and Radiation. *Emissions Modeling Platforms*. Air Emissions Modeling. https://gaftp.epa.gov/Air/nei/2017/doc/flat_files/ (accessed 2020-05-20).

(15) NOAA's Physical Sciences Laboratory. *NCEP-NCAR Reanalysis 1*. Gridded Climate Data: NCEP/NCAR Reanalysis. https://psl.noaa.gov/data/gridded/data.ncep.reanalysis.html (accessed 2022-04-27).

(16) Dennin, L. R.; Nock, D.; Muller, N. Z.; Akindele, M.; Adams, P. J. Modeling Wildland Fire Smoke Damages in the U.S. and Unpacking Impact Disparities by Social Vulnerability. *Manuscript Under Review* **2024**.

(17) Turner, D. B. 2: Estimates of Atmospheric Dispersion. In *Workbook of Atmospheric Dispersion Estimates: An Introduction to Dispersion Modeling*; CRC Press, Inc.: Boca Raton, FL, 1994; p 2.1-2.38.

(18) Ramaswami, A.; Milford, J. B.; Small, M. J. Models of Transport in Air. In *Integrated Environmental Modeling: Pollutant Transport, Fate, and Risk in the Environment*; John Wiley & Sons, Inc.: Hoboken, NJ, 2005; pp 280–314.
(19) Seinfeld, J. H.; Pandis, S. N. Atmospheric Diffusion. In *Atmospheric Chemistry and Physics: From Air Pollution to Climate Change*; John Wiley & Sons, Inc.: Hoboken, NJ, 2016; pp 763–828.
(20) Turner, D. B. 4: Special Topics. In *Workbook of Atmospheric Dispersion Estimates: An Introduction to Dispersion Modeling*; CRC Press, Inc.: Boca Raton, FL, 1994; p 4.1-4.29.
(21) Xu, G.; Zhang, Q.; Yao, Y.; Zhang, X. Changes in PM2.5 Sensitivity to NOx and NH3 Emissions Due to a Large Decrease in SO2 Emissions from 2013 to 2018. *Atmospheric and Oceanic Science Letters* **2020**, *13* (3), 210–215. https://doi.org/10.1080/16742834.2020.1738009.
(22) Saylor, R.; Myles, L.; Sibble, D.; Caldwell, J.; Xing, J. Recent Trends in Gas-Phase Ammonia and PM2.5 Ammonium in the Southeast United States. *Journal of the Air & Waste Management Association* **2015**, *65* (3), 347–357. https://doi.org/10.1080/10962247.2014.992554.
(23) Tsimpidi, A. P.; Karydis, V. A.; Pandis, S. N. Response of Inorganic Fine Particulate Matter to Emission Changes of Sulfur Dioxide and Ammonia: The Eastern United States as a Case Study. *Journal of the Air & Waste Management Association* **2007**, *57* (12), 1489–1498. https://doi.org/10.3155/1047-3289.57.12.1489.
(24) U.S. EPA's Clean Air Technology Center. *Nitrogen Oxides (NOx), Why and How They Are Controlled*; EPA-456/F-99-006R; U.S. Environmental Protection Agency: Research Triangle Park, NC, 1999. https://www3.epa.gov/ttncatc1/dir1/fnoxdoc.pdf.
(25) Jaramillo, P.; Muller, N. Z. Air Pollution Emissions and Damages from Energy Production in the U.S.: 2002–2011. *Energy Policy* **2016**, *90*, 202–211. https://doi.org/10.1016/j.enpol.2015.12.035.
(26) U.S. EPA's Office of Air and Radiation. *2020 National Emissions Inventory (NEI) Data*. Air Emissions Inventories. https://www.epa.gov/air-emissions-inventories/2020-national-emissions-inventory-nei-data (accessed 2023-03-07).
(27) Yu, F.; Nair, A. A.; Luo, G. Long-Term Trend of Gaseous Ammonia Over the United States: Modeling and Comparison With Observations. *J Geophys Res Atmos* **2018**, *123* (15), 8315–8325. https://doi.org/10.1029/2018JD028412.
(28) Hernandez, C. Decision-Making Tools for the Mitigation of Particulate Matter Health Impacts. thesis, Carnegie Mellon University, 2023. https://doi.org/10.1184/R1/22321054.v1.
(29) Wise, M.; Calvin, K.; Thomson, A.; Clarke, L.; Bond-Lamberty, B.; Sands, R.; Smith, S. J.; Janetos, A.; Edmonds, J. Implications of Limiting CO2 Concentrations for Land Use and Energy. *Science* **2009**, *324* (5931), 1183–1186. https://doi.org/10.1126/science.1168475.
(30) Smith, S. J.; Wigley, T. M. L. Multi-Gas Forcing Stabilization with Minicam. *The Energy Journal* **2006**, *27* (3_suppl), 373–392. https://doi.org/10.5547/ISSN0195-6574-EJ-VolSI2006-NoSI3-19.
(31) Grubert, E. Emissions Projections for US Utilities through 2050. *Environ. Res. Lett.* **2021**, *16* (8), 084049. https://doi.org/10.1088/1748-9326/ac1628.
(32) Campbell, P.; Zhang, Y.; Yan, F.; Lu, Z.; Streets, D. Impacts of Transportation Sector Emissions on Future U.S. Air Quality in a Changing Climate. Part I: Projected Emissions,

Simulation Design, and Model Evaluation. *Environmental Pollution* **2018**, *238*, 903–917. https://doi.org/10.1016/j.envpol.2018.04.020.
(33) Sutton, M. A.; Reis, S.; Riddick, S. N.; Dragosits, U.; Nemitz, E.; Theobald, M. R.; Tang, Y. S.; Braban, C. F.; Vieno, M.; Dore, A. J.; Mitchell, R. F.; Wanless, S.; Daunt, F.; Fowler, D.; Blackall, T. D.; Milford, C.; Flechard, C. R.; Loubet, B.; Massad, R.; Cellier, P.; Personne, E.; Coheur, P. F.; Clarisse, L.; Van Damme, M.; Ngadi, Y.; Clerbaux, C.; Skjøth, C. A.; Geels, C.; Hertel, O.; Wichink Kruit, R. J.; Pinder, R. W.; Bash, J. O.; Walker, J. T.; Simpson, D.; Horváth, L.; Misselbrook, T. H.; Bleeker, A.; Dentener, F.; de Vries, W. Towards a Climate-Dependent Paradigm of Ammonia Emission and Deposition. *Philos Trans R Soc Lond B Biol Sci* **2013**, *368* (1621), 20130166. https://doi.org/10.1098/rstb.2013.0166.
(34) Malley, C. S.; Hicks, W. K.; Kulyenstierna, J. C. I.; Michalopoulou, E.; Molotoks, A.; Slater, J.; Heaps, C. G.; Ulloa, S.; Veysey, J.; Shindell, D. T.; Henze, D. K.; Nawaz, O.; Anenberg, S. C.; Mantlana, B.; Robinson, T. P. Integrated Assessment of Global Climate, Air Pollution, and Dietary, Malnutrition and Obesity Health Impacts of Food Production and Consumption between 2014 and 2018. *Environ. Res. Commun.* **2021**, *3* (7), 075001. https://doi.org/10.1088/2515-7620/ac0af9.
(35) Domingo, N. G. G.; Balasubramanian, S.; Thakrar, S. K.; Clark, M. A.; Adams, P. J.; Marshall, J. D.; Muller, N. Z.; Pandis, S. N.; Polasky, S.; Robinson, A. L.; Tessum, C. W.; Tilman, D.; Tschofen, P.; Hill, J. D. Air Quality–Related Health Damages of Food. *Proceedings of the National Academy of Sciences* **2021**, *118* (20), e2013637118. https://doi.org/10.1073/pnas.2013637118.
(36) Gu, B.; Zhang, L.; Van Dingenen, R.; Vieno, M.; Van Grinsven, H. J.; Zhang, X.; Zhang, S.; Chen, Y.; Wang, S.; Ren, C.; Rao, S.; Holland, M.; Winiwarter, W.; Chen, D.; Xu, J.; Sutton, M. A. Abating Ammonia Is More Cost-Effective than Nitrogen Oxides for Mitigating PM2.5 Air Pollution. *Science* **2021**, *374* (6568), 758–762. https://doi.org/10.1126/science.abf8623.
(37) Sergi, B. J. Integrating Climate and Health Damages in Decision-Making for the Electric Power Sector. thesis, Carnegie Mellon University, 2019. https://doi.org/10.1184/R1/8204723.v1.
(38) Ramboll. *A multi-scale photochemical modeling system for gas and particulate air pollution: Comprehensive Air Quality Model with Extensions*. CAMx. http://www.camx.com/home/ (accessed 2023-01-10).
(39) Jerrett, M.; Burnett, R. T.; Pope, C. A.; Ito, K.; Thurston, G.; Krewski, D.; Shi, Y.; Calle, E.; Thun, M. Long-Term Ozone Exposure and Mortality. *N Engl J Med* **2009**, *360* (11), 1085–1095. https://doi.org/10.1056/NEJMoa0803894.
(40) Turner, M. C.; Jerrett, M.; Pope, C. A.; Krewski, D.; Gapstur, S. M.; Diver, W. R.; Beckerman, B. S.; Marshall, J. D.; Su, J.; Crouse, D. L.; Burnett, R. T. Long-Term Ozone Exposure and Mortality in a Large Prospective Study. *Am J Respir Crit Care Med* **2016**, *193* (10), 1134–1142. https://doi.org/multi.
(41) U.S. EPA's Office of Air and Radiation. *Air Quality System (AQS): Pregenerated Data Files*. Air Data: Air Quality Data Collected at Outdoor Monitors Across the US. https://aqs.epa.gov/aqsweb/airdata/download_files.html (accessed 2022-11-21).
(42) Tschofen, P.; Muller, N. Z.; Dennin, L. R.; Azevedo, I. L. Climate, Air, and Environmental Damages Call for a Reevaluation of Regulatory Priorities for Emissions in the United States. *Manuscript in Preparation* **2023**.

(43) Tschofen, P. Essays in Pollution Accounting. Ph.D., Carnegie Mellon University, 2023. https://www.proquest.com/docview/2810802384/abstract/87EAA1C075C54D49PQ/1 (accessed 2023-09-15).
(44) Sergi, B. J.; Azevedo, I.; Davis, S. J.; Muller, N. Z. Regional and County Flows of Particulate Matter Damage in the US. *Environ. Res. Lett.* **2020**, *15* (10), 104073. https://doi.org/10.1088/1748-9326/abb429.
(45) Boylan, J. W.; Russell, A. G. PM and Light Extinction Model Performance Metrics, Goals, and Criteria for Three-Dimensional Air Quality Models. *Atmospheric Environment* **2006**, *40* (26), 4946–4959. https://doi.org/10.1016/j.atmosenv.2005.09.087.
(46) Morris, R. E.; McNally, D. E.; Tesche, T. W.; Tonnesen, G.; Boylan, J. W.; Brewer, P. Preliminary Evaluation of the Community Multiscale Air Quality Model for 2002 over the Southeastern United States. *Journal of the Air & Waste Management Association* **2005**, *55* (11), 1694–1708. https://doi.org/10.1080/10473289.2005.10464765.
(47) Tessum, C. W.; Hill, J. D.; Marshall, J. D. InMAP: A Model for Air Pollution Interventions. *PLoS ONE* **2017**, *12* (4), e0176131. https://doi.org/10.1371/journal.pone.0176131.
(48) Heo, J.; Adams, P. J.; Gao, H. O. Reduced-Form Modeling of Public Health Impacts of Inorganic PM2.5 and Precursor Emissions. *Atmospheric Environment* **2016**, *137*, 80–89. https://doi.org/10.1016/j.atmosenv.2016.04.026.
(49) Muller, N. Z.; Mendelsohn, R.; Nordhaus, W. Environmental Accounting for Pollution in the United States Economy. *The American Economic Review* **2011**, *101* (5), 1649–1675.
(50) Krewski, D.; Jerrett, M.; Burnett, R. T.; Ma, R.; Hughes, E.; Shi, Y.; Turner, M. C.; Pope, C. A.; Thurston, G.; Calle, E. E.; Thun, M. J.; Beckerman, B.; DeLuca, P.; Finkelstein, N.; Ito, K.; Moore, D. K.; Newbold, K. B.; Ramsay, T.; Ross, Z.; Shin, H.; Tempalski, B. Extended Follow-up and Spatial Analysis of the American Cancer Society Study Linking Particulate Air Pollution and Mortality. *Res Rep Health Eff Inst* **2009**, No. 140, 5–114; discussion 115-136.
(51) Di, Q.; Wang, Y.; Zanobetti, A.; Wang, Y.; Koutrakis, P.; Choirat, C.; Dominici, F.; Schwartz, J. D. Air Pollution and Mortality in the Medicare Population. *N Engl J Med* **2017**, *376* (26), 2513–2522. https://doi.org/10.1056/NEJMoa1702747.
(52) CDC. *National Center for Health Statistics Mortality Data on CDC WONDER*. CDC WONDER. https://wonder.cdc.gov/mcd.html (accessed 2023-10-12).
(53) CDC. *Bridged-Race Population Estimates*. CDC WONDER. https://wonder.cdc.gov/bridged-race-population.html (accessed 2023-10-12).
